# Role of Gemcitabine and Pemetrexed as Maintenance Therapy in Advanced NSCLC: A Systematic Review and Meta-Analysis of Randomized Controlled Trials

**DOI:** 10.1371/journal.pone.0149247

**Published:** 2016-03-08

**Authors:** Xingsheng Hu, Ke Pu, Xuqin Feng, Shimin Wen, Xi Fu, Cuihua Guo, Wenwu He

**Affiliations:** 1 Department of Oncology, Nanchong Central Hospital (The Second Clinical College of North Sichuan Medical College), Nanchong, China; 2 DaZhou College of Chinese Medicine, DaZhou, China; 3 Department of Cardiothoracic Surgery, Nanchong Central Hospital, Nanchong, China; Catalan Institute of Oncology, SPAIN

## Abstract

**Background:**

Gemcitabine and pemetrexed have been used as maintenance therapy. However, few systematic reviews and meta-analyses have assessed their effects in the newest studies. This systematic review and meta-analysis were conducted to assess the role of gemcitabine and pemetrexed in the maintenance treatment of non-small-cell lung carcinoma (NSCLC).

**Methods:**

We performed a literature search using PubMed, EMBASE and Cochrane library databases from their inceptions to September 16, 2015. We also searched the American Society of Clinical Oncology (ASCO), European Society for Medical Oncology (ESMO), and National Comprehensive Cancer Network (NCCN) databases from 2008 to 2015. Two authors independently extracted the data. The Cochrane Collaboration’s risk of bias graph was used to assess the risk of bias. The GRADE system was used to assess the grading of evidence, and a meta-analysis was conducted using Stata 11.0 software.

**Results:**

Eleven randomized controlled trial (RCT) studies were collected. Ten studies were included in the meta-analysis and divided into the following 4 groups: gemcitabine vs. best supportive care (BSC)/observation, pemetrexed vs. BSC/placebo, pemetrexed + bevacizumab vs. bevacizumab and pemetrexed vs. bevacizumab. Gemcitabine exhibited significantly improved progression-free survival (PFS) compared with BSC (hazard ratio (HR) = 0.62, p = 0.000). Pemetrexed exhibited significantly improved PFS (HR = 0.54, p = 0.000) and OS (HR = 0.75, p = 0.000) compared with BSC. Pemetrexed + bevacizumab almost exhibited significantly improved PFS (HR = 0.71, p = 0.051) compared with bevacizumab. Pemetrexed exhibited no improvement in PFS or overall survival (OS) compared with bevacizumab. Regarding the grade, the GRADE system indicated that the gemcitabine group was "MODERATE", the pemetrexed group was "HIGH", and both the pemetrexed + bevacizumab vs. bevacizumab groups and pemetrexed vs. B groups were "LOW".

**Conclusions:**

Gemcitabine or pemetrexed compared with BSC/observation/placebo significantly improved PFS or OS. Whether pemetrexed + bevacizumab compared with bevacizumab alone significantly improves PFS requires further investigation.

## Introduction

Lung cancer is the leading cancer in both incidence and mortality and accounts for 25% of all cancer deaths [1]. Additionally, the incidence of lung cancer is increasing in some regions. Non-small-cell lung carcinoma (NSCLC) accounts for greater than 80% of all lung cancers. In the past decades, the standard first-line treatment for advanced NSCLC consisted of platinum-based doublet therapy for no more than six cycles [2]. However, there is generally a brief period of disease control after the response to first-line chemotherapy, and most of patients will die because of disease progression. Thus, the 5-year survival rate is very low (less than 5%) [3, 4, 5]. Consequently, it is necessary to identify more effective and tolerable treatments to delay progression and improve survival in advanced-stage NSCLC.

Maintenance therapy is one strategy that has been investigated extensively in recent years. Currently, only two chemotherapy agents have been recommended for advanced NSCLC by National Comprehensive Cancer Network (NCCN) guidelines, gemcitabine and pemetrexed. Several RCTs have demonstrated that gemcitabine [6, 7] or pemetrexed [8, 9, 10] compared with BSC/placebo improves PFS and that pemetrexed improves OS more effectively. However, few systematic reviews or meta-analyses have analyzed these newest RCTs. In his meta-analysis, Behera [11] pooled different therapeutic approaches and incorporated the overall HR for gemcitabine, pemetrexed, and other chemotherapy agents, such as epidermal growth factor receptor-tyrosine kinase inhibitors (EGFR-TKIs). Soon [12] also indiscriminatingly mixed different maintenance treatment agents, including different first-line chemotherapy programs, to incorporated an overall HR. Apparently, these analyses were not accurate or objective, confusing the efficiency of the single therapeutic agents. Regarding gemcitabine, Zhang [13] conducted a meta-analysis including three gemcitabine trials (Brodowicz [6], Belani [14] and Perol [15] (the Perol [15] trial was only an abstract)), and the data were not mature. Regarding pemetrexed, Qi [16] conducted a meta-analysis of pemetrexed vs. placebo to assess PFS and only included two studies (Ciuleanu [8] and Paz-Ares [17] (the Paz-Ares study was only an abstract)), and the OS data were not mature. Thus, a meta-analysis for OS comparison was not conducted. In addition, in the recent 3 years, other evidence of pemetrexed maintenance therapy has emerged. Pemetrexed + bevacizumab compared with bevacizumab alone improves PFS but did not improve OS [18, 19, 20]. Therefore, there is a great need to conduct a systematic review and meta-analysis to assess these up-to-date studies.

In this systematic review and meta-analysis, we updated the Perol (2010) study [15] to Perol (2012) [7] as well as Paz-Ares (2011) [17] to Paz-Ares (2012[9] /2013[10]) by pooling the pemetrexed ± bevacizumab vs. bevacizumab analyses, and collected data from other studies on pemetrexed vs. docetaxel in maintenance therapy. More importantly, we used the Cochrane Collaboration tool to assess the risk of bias and the GRADE system to assess the grade of evidence.

## Materials and Methods

### Study design

This systematic review and meta-analysis strictly followed the Preferred Reporting Items for Systematic Reviews and Meta-Analyses (PRISMA) statement guidelines 2009 [21]. Except for Brodowic [6], Belani [14] and Karayama [22] studies, all of the other studies have protocols, which were available from https://clinicaltrials.gov.

### Eligibility criteria

The following study selection criteria were applied: (1) population: patients were pathologically diagnosed with advanced chemotherapy-naïve NSCLC; (2) intervention: gemcitabine or pemetrexed as a single agent was applied in maintenance therapy after 4 to 6 cycles of induction chemotherapy; (3) comparison: no restrictions were imposed and included BSC/observation, cytotoxic agents, vascular endothelial growth factor receptor (VEGFR), EGFR-TKI or any other therapeutic drugs; (4) outcomes: HR of PFS and OS, risk ratios (RR) of grade 3–4 adverse events (AEs); (5) study design: only RCTs were eligible.

### Literature search

Electronic databases, including PubMed, EMBASE, and Cochrane Central Register of Controlled Trials (CENTRAL), were searched for relevant clinical trials published from their inceptions to September 16, 2015. The following key words were applied: (1) “lung cancer gemcitabine maintenance” and (2) “lung cancer pemetrexed maintenance”. After the first search, article types were chosen as follows: "clinical trial" was chosen in PubMed, "randomized control trials" was chosen in EMBASE, and no restrictions were imposed in the Cochrane library. Additionally, no language restrictions were imposed. Furthermore, we screened the references from the retrieved original articles and screened the ASCO, ESMO, and NCCN databases between 2008 and 2015 to identify any other potentially eligible studies.

### Study selection

The selection of trials main was accorded to eligibility criteria. This process were performed by two authors and blinded. The meeting abstracts fulfilling the criteria were also included. The references were screened by titles and further selected by reading the abstracts.

### Data extraction and items

Two reviewers independently extracted the following data from each eligible study: first author’s last name and year of publication, trial’s name and registration number, number of patients, region and race, histology, the drugs of induction and maintenance therapy, HR of PFS and OS, and the incidence of grade 3–4 AEs. Any disagreements were resolved by consensus or consultation with a third reviewer.

### Assessing the risk of bias and grading the quality of evidence

According to the new Cochrane handbook (version 5.1.0), which no longer recommends any quality assessment tables or checklists to assess the quality of original articles, the Cochrane Collaboration’s tool was adopted to assessing the risk of bias [23], and the GRADE system was used to assess the grades of evidence[24].

The assessment for the risk of bias was strictly performed according to the guidelines outlined in the Cochrane handbook. Two investigators objectively reviewed all of the studies and assigned a value of ‘‘low”, ‘‘unclear” or “high” to the following six domains: random sequence generation, allocation concealment, blinding of participants and personnel, blinding of outcome assessment, incomplete outcome data, selective reporting, and other bias. All of the open-label trials were judged as "high risk" in the blinding of participants (performance bias) and researchers as well as blinding of the outcome assessment (detection bias).

The GRADE system identified the following four grades to rate the quality of evidence [25]: (1) high: further research is very unlikely to change the estimate of the effect; (2) moderate: further research is likely to impact the estimate of the effect and may change the estimate; (3) low: further research is very likely to impact the estimate of the effect and is likely to change the estimate; and (4) very low: any estimate of the effect is very uncertain.

### Statistical analysis

We estimated HRs and 95% confidence intervals (CIs) for PFS and OS and the RR for the grade 3–4 AEs. Heterogeneity was determined using the chi-squared-based Cochran’s Q statistic and I^2^ statistic. I^2^ values of 0–40%, 40–70% and 70–100% were used to represent low, moderate and high variance, respectively [26]. If moderate heterogeneity existed or different clinical characteristics were noted, the random-effects model was used. Otherwise, the fixed-effects model was used. If significant heterogeneity was identified, subgroup analysis or sensitivity analyses were conducted. Potential publication bias was evaluated by funnel plots and Egger’s weighted linear regression test. RevMan 5.3 was used to generate the figure of the "Cochrane Collaboration’s tool for assessing risk of bias". The GRADE profiler software (version 3.6) (available at: http://www.grade/workinggroup.org/) was used to assess the grades of evidence. All of the other statistical data analyses were performed using Stata 11.0. All of the p-values were two-sided and were considered statistically significant at the 0.05 level.

## Results

### Study selection and characteristics

**Fig 1:**
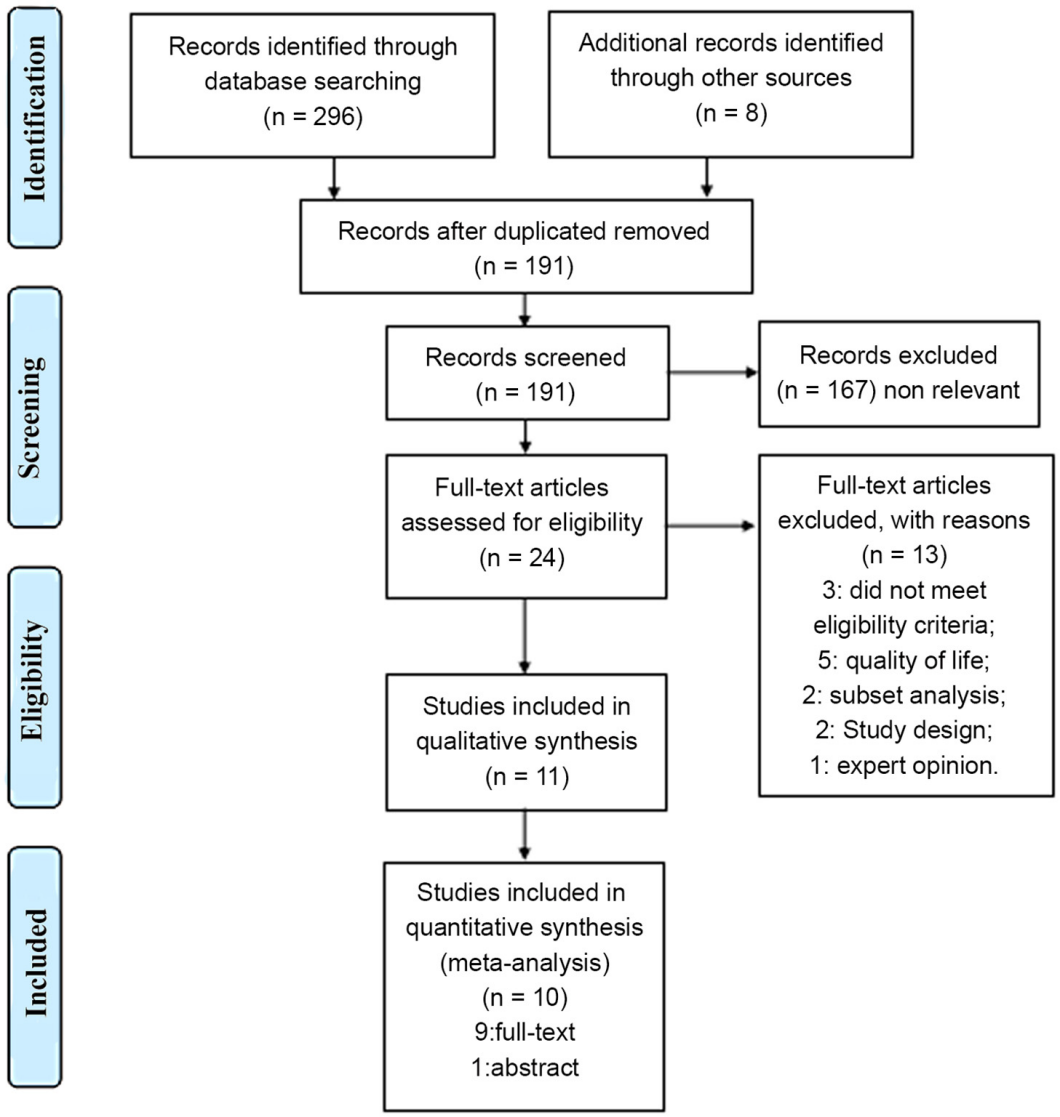
Flow diagram of the details of the study.

Three hundred four relevant citations were identified at the initial search stage. Finally, 11 studies were included in this systematic review, and 10 studies were included in the meta-analysis. These studies were divided into the following 4 groups: gemcitabine vs. BSC/observation, pemetrexed vs. BSC±placebo, pemetrexed +bevacizumab vs. bevacizumab, and pemetrexed vs. bevacizumab. Other studies concerning pemetrexed vs. docetaxel were qualitatively analyzed separately. The flow diagram of the literature retrieval and selection is presented in Fig 1.

The main characteristics of all of the eligible RCTs are presented in Tables 1 and 2. Except for the Mubarak [27] and Karayama [22] were multicenter phase II clinical trials, all of the other studies were multicenter phase III clinical trials. The Ciuleanu [8] and Paz-Ares [9, 10] studies involved randomized, double-blind trials, whereas the Perol [7], Mubarak [26], Patel [20], Barlesi [18, 19], Zinner [28], Galetta [29] and Karayama [29] studies were randomized, open-label trials. Only the Brodowicz[6] trial did not describe whether it was a double-blind or open-label trial.

**Table 1:** Main characteristics of the studies.

study	study name, registration number	pts	region and Race	Histology	Induction treatment	Maintenance treatment	HR of PFS, exp VS control	HR of OS, exp VS control
Brodowicz2006[6]	CECOG	206	Europe	NSCLC	GC	G+BSC VS BSC	0.66(0.56–0.85)a	0.84(0.51–1.36)a
			White				p<0.001	p = 0.195
Belani2010[14]	—	255	USA,	NSCLC	GC	G+BSC VS BSC	-	0.97(0.72–1.30)a
			NA					p = 0.84
Perol2012[7]	IFCT-GFPC	464	France,	NSCLC	GC	G VS observation	0.56(0.44–0.72)a	0.89(0.69–1.15)a
	0502		White				P<0.001	p = 0.3867
	NCT00300586							
Ciuleanu2009[8]	JMEN	663	Europe, Asian	NSCLC	GC, PaC,	P+BSC VS	0.50(0.42–0.61)a	0.79(0.65–0.95)a
	NCT00102804		White 65%,		DC	Placebo+ BSC	p<0.0001	p = 0.012
			Asian 32%					
		481		nonsquamous			0.44(0.36–0.55)a	0.70(0.56–0.88)a
							p<0.0001	p = 0.002
Paz-Ares2012[9, 10]	PARAMOUNT	539	Europe	nonsquamous	PC	P+BSC VS	0.62(0.49–0.79)a	0.78(0.64–0.96)a
	NCT00789373		White 94.6%			Placebo+BSC	p<0.0001	p = 0.0195
Mubarak2012[27]	NCT00606021	55	Middle East	nonsquamous	PC	P + BSC	0.65(0.35–1.20)a	0.95(0.46–1.97)a
			White 94.5%			vs BSC	p = 0.084	p = 0.4497
Barlesi2014[18, 19]	AVAPERL	376	Europe	nonsquamous	PC+B	P+B VS B	0.58(0.45–0.76)b	0.88(0.64–1.22)b
	NCT00961415		White				P<0.0001	P<0.32
Patel2013[20]	PointBreak	939	USA,	nonsquamous	PC+B VS PaC+B	P+B VS B	0.83(0.71–0.96)b	1.00(0.86–1.16)b
	NCT00762034		White 85.7%				p = 0.012	p = 0.949
			black 10.0%					
Zinner2015[28]	PRONOUNCE	361	USA,	nonsquamous	PC VS PaC+B	P VS B	1.06(0.84–1.35)b	1.07(0.83–1.36)b
	NCT00948675		White 89.2%				p = 0.610	p = 0.615
			black 8.6%					
Galetta2015[29]	ERACLE	118	Italy,	nonsquamous	PC VS PaC+B	P VS B	0.79(0.53–1.17)b	0.93(0.60–1.42)b
	NCT01303926		White				p = 0.24	p = 0.73
Karayama2013[22]	UMIN ID	51	Japan,	nonsquamous	PC	P VS D	control VS exp	exp VS control
	000004075		Asian				0.56(0.28–1.08)a	0.79(0.32–2.00)a
							p = 0.084	p = 0.622

Pts: patients; GC: gemcitabine+cisplatin; PaC: paclitaxel+cisplati; DC: docetaxel+cisplatin; PC: pemetrexed+cisplatin; B: bevacizumab; D: docetaxel; BSC: best supportive care; exp: experiment; VS: versus; a: time from maintenance treatment; b:time from induction treatment.

**Table 2:** The incidence of grade 3–4 AEs.

study	positive	negative	positive	negative
Belani2010	32	96	9	118
Perol2012	64	90	11	144
Ciuleanu2009	70	371	9	213
Paz-Ares2012	131	228	13	167
Mubarak2012	4	24	4	23
Barlesi2014	102	23	71	49
Patel2013	366	76	310	133
Zinner2015	117	54	121	45
Galetta2015	13	47	22	36

### Risk of bias and grades of evidence

**Fig 2:**
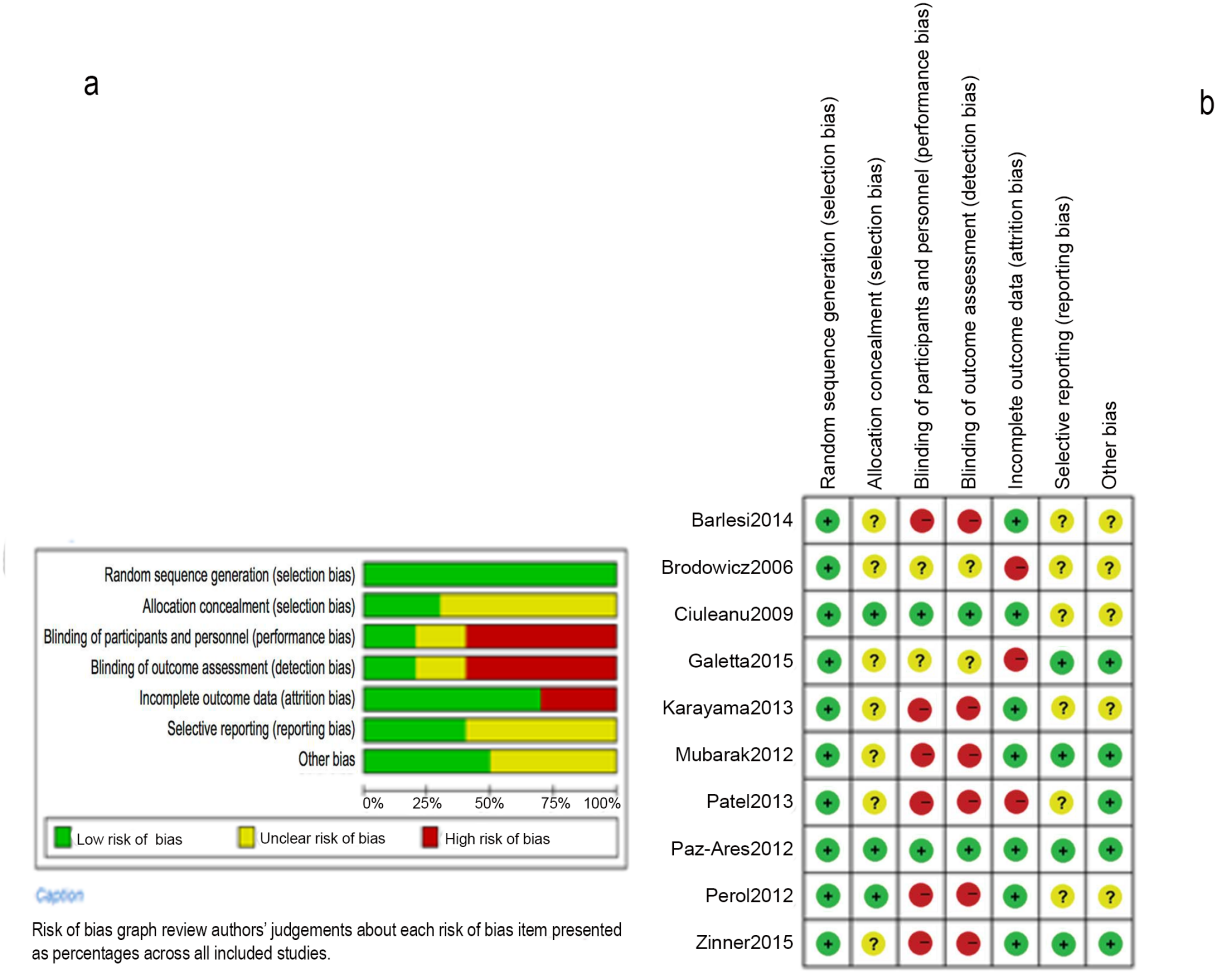
Risk of bias graph (a) and risk of bias summary (b).

The results for assessing the risk of bias are shown in Fig 2, and the grades of evidence are presented in Tables 3–6. Two double-blind trials offered better descriptions of random sequence generation, allocation concealment, blinding of participants and personnel, and blinding of outcome assessment. All of the open-label trials did not describe the details of allocation concealment, and, more importantly, their main bias was the lack of blinding.

**Table 3:** GRADE profile evidence of the included studies for gemcitabine VS BSC/observation.

Quality assessment							No of patients		Effect		Quality	Importance
No of studies	Design	Risk of bias	Inconsistency	Indirectness	Imprecision	Other considerations	Pem	BSC	Relative (95% CI)	Absolute		
progression free survival (follow-up median 20.5–25.6 months; assessed with: follow up)												
2	randomised trials	serious^1^	no serious inconsistency	no serious indirectness	no serious imprecision	none	0/292 (0%)	0/223 (0%)	HR 0.62 (0.53 to 0.72)	-	MODERATE	CRITICAL
overall survival (follow-up median 20.5–25.6 months; assessed with: follow up)												
3	randomised trials	serious^1^	no serious inconsistency	no serious indirectness	no serious imprecision	none	0/420 (0%)	0/350 (0%)	HR 0.91 (0.76 to 1.09)	-	MODERATE	CRITICAL
grade 3–4 adverse events (follow-up median 25.6 months; assessed with: observation)												
2	randomised trials	serious^1^	no serious inconsistency	no serious indirectness	no serious imprecision	very strong association^2^	96/282 (34%)	20/282 (7.1%)	RR 4.70 (2.87 to 7.69)	262 more per 1000 (from 133 more to 474 more)	HIGH	IMPORTANT

^1^ 3 study excited bias in allocation concealment and blinding

^2^ 1 study show RR>2,and another study show RR>5.

**Table 4:** GRADE profile evidence of the included studies for pemetrexed VS BSC/placebo.

Quality assessment							No of patients		Effect		Quality	Importance
No of studies	Design	Risk of bias	Inconsistency	Indirectness	Imprecision	Other considerations	Pem	BSC	Relative (95% CI)	Absolute		
progression free survival (follow-up median 11.2–12.5 months; assessed with: follow up)												
3	randomised trials	no serious risk of bias	no serious inconsistency	no serious indirectness	no serious imprecision	increased effect for RR ~1^2^	0/712 (0%)	0/363 (0%)	-	-	HIGH	CRITICAL
overall survival (follow-up median 11.2–12.5 months; assessed with: follow up)												
3	randomised trials	no serious risk of bias	no serious inconsistency	no serious indirectness	no serious imprecision	increased effect for RR ~1^2^	0/712 (0%)	0/363 (0%)	RR 0.75 (0.65 to 0.87)	-	HIGH	CRITICAL
grade 3–4 adverse events (follow-up median 11.2–12.5 months; assessed with: observation)												
3	randomised trials	no serious risk of bias	serious^1^	no serious indirectness	no serious imprecision	very strong association^3^ reduced effect for RR >> 1 or RR << 1	205/828 (24.8%)	26/429 (6.1%)	RR 3.27(1.56 to 6.83)	138 more per 1000 (from 34 more to 353 fewer)	HIGH	IMPORTANT

^1^ 1 study inconsistency.

^2^ if squamous histology, the HR will increase

^3^ 1 study show RR>2, and another study show RR>5.

**Table 5:** GRADE profile evidence of the included studies for pemetrexed+bevacizumab VS bevacizumab.

Quality assessment							No of patients		Effect		Quality	Importance
No of studies	Design	Risk of bias	Inconsistency	Indirectness	Imprecision	Other considerations	Pem	B	Relative (95% CI)	Absolute		
progression free survival (follow-up median 11.9–14.8 months; assessed with: follow up)												
2	randomised trials	serious^1^	serious^2^	no serious indirectness	no serious imprecision	none	0/567 (0%)	0/563 (0%)	HR 0.71 (0.50 to 1.00)	-	LOW	CRITICAL
overall survival (follow-up median11.9–14.8 months; assessed with: follow up)												
2	randomised trials	serious^1^	no serious indirectness	no serious indirectness	no serious imprecision	none	0/567 (0%)	0/563 (0%)	HR 0.98 (0.85 to 1.12)	-	MODERATE	CRITICAL
"grade 3–4 adverse events" (follow-up median 11.9–14.8 months; assessed with: observation)												
2	randomised trials	serious^1^	serious^2^	no serious indirectness	no serious imprecision	none	468/567 (82.5%)	381/563 (67.7%)	RR1.25(1.08 to1.45)	169 more per 1000 (from 54 more to 305 fewer)	LOW	IMPORTANT

^1^ 2 study excited bias in allocation concealment and blinding.

^2^ 2 study excited large heterogeneity.

**Table 6:** GRADE profile evidence of the included studies for pemetrexed VS bevacizumab.

Quality assessment							No of patients		Effect		Quality	Importance
No of studies	Design	Risk of bias	Inconsistency	Indirectness	Imprecision	Other considerations	Pem	B	Relative (95% CI)	Absolute		
progression free survival (follow-up median 27.0 months; assessed with: follow up)												
2	randomised trials	serious^1^	no serious indirectness	no serious indirectness	no serious imprecision	none	0/231 (0%)	0/224 (0%)	HR 0.96 (0.73 to 1.26)	—	MODERATE	CRITICAL
overall survival (follow-up median 27 months; assessed with: follow up)												
2	randomised trials	serious^1^	no serious indirectness	no serious indirectness^2^	no serious imprecision	none	0/231 (0%)	0/224 (0%)	HR 1.03 (0.83 to 1.28)	—	MODERATE	CRITICAL
"grade 3–4 adverse events" (follow-up median 27.0 months; assessed with: observation)												
2	randomised trials	serious^1^	serious^2^	no serious indirectness	no serious imprecision	none	130/231 (56.3%)	143/224 (63.8%)	RR0.79(0.49 to1.29)	134 fewer per 1000 (from 326 fewer to185fewer)	LOW	IMPORTANT

^1^ 2 study excited bias in allocation concealment and blinding.

^2^ 2 study excited large heterogeneity.

### PFS

**Fig 3:**
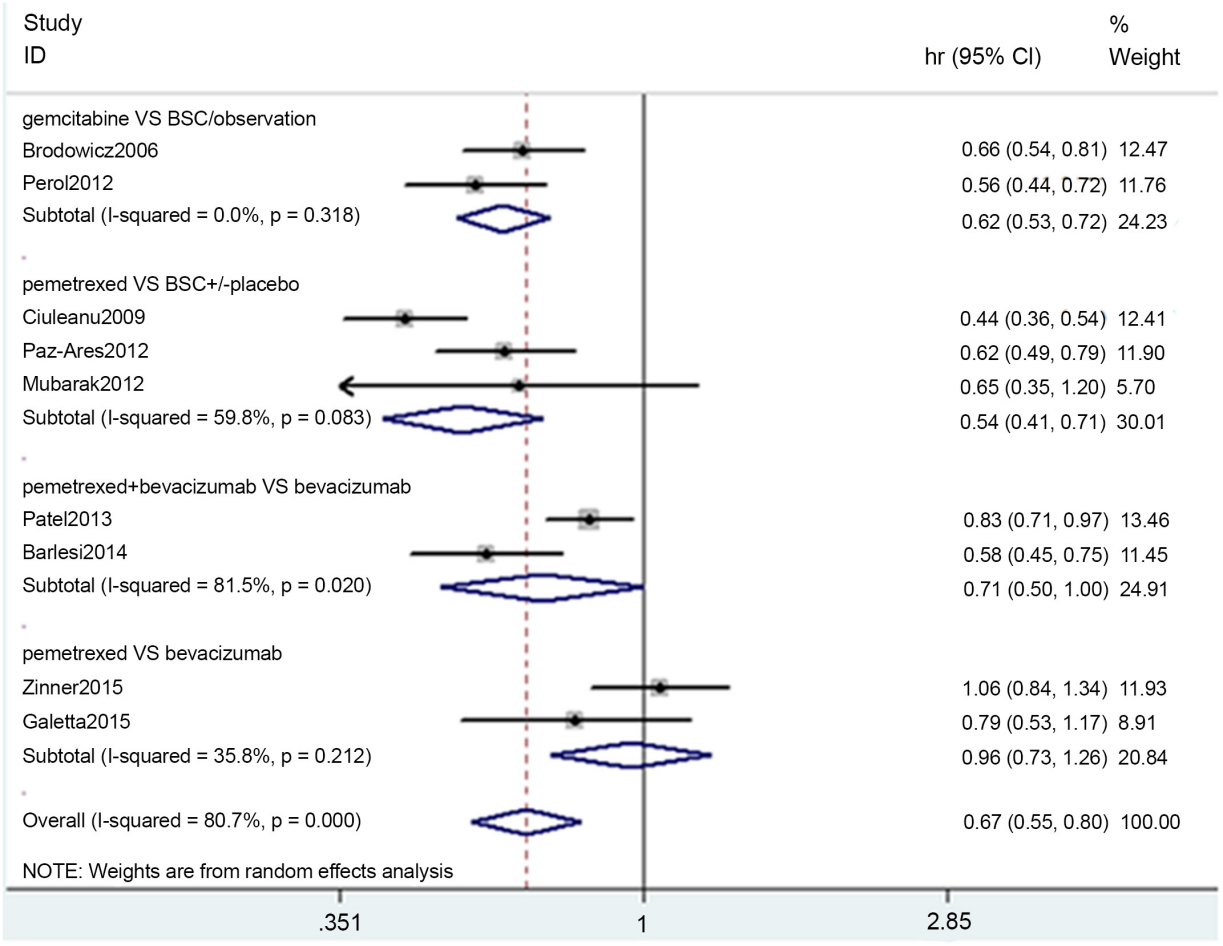
Meta-analysis results of PFS.

The meta-analysis pooled results are presented in Fig 3. The heterogeneity test indicated that a random-effects model could be selected. As a result, in the gemcitabine vs. BSC/observation group, the pooled HR was 0.62 (0.53–0.72, p = 0.000; I^2^ = 0.0%, p = 0.318). In the pemetrexed vs. BSC ± placebo group, the pooled HR was 0.54 (0.41–0.71, p = 0.000; I^2^ = 59.8%, p = 0.083). In the pemetrexed+ bevacizumab vs. bevacizumab group, the HR was 0.71 (0.50–1.00, p = 0.051; I^2^ = 81.5%, p = 0.02). In the pemetrexed vs. bevacizumab group, the HR was 0.96 (0.73–1.26, p = 0.752; I^2^ = 35.8%, p = 0.212).

### OS

**Fig 4:**
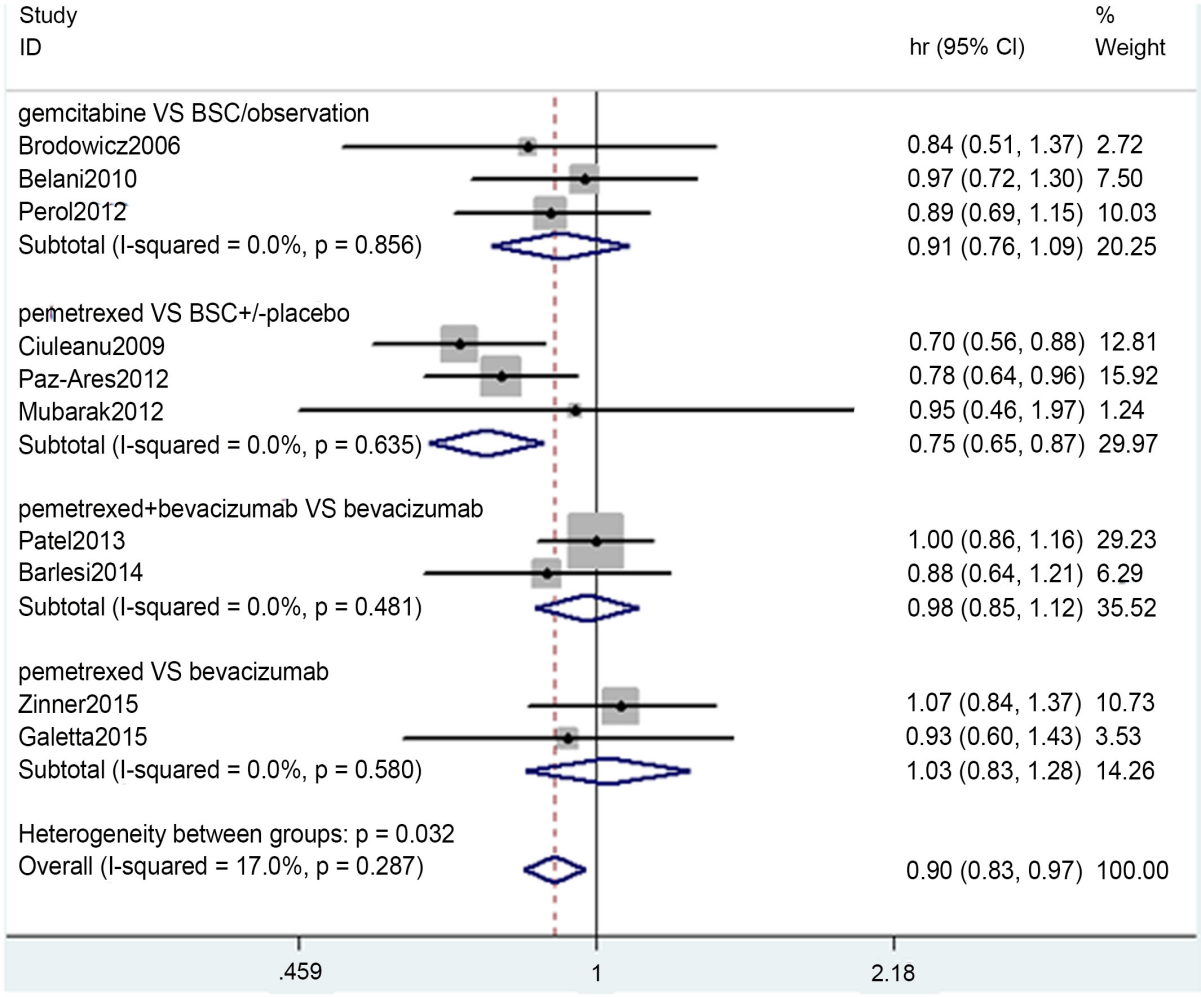
Meta-analysis results of OS.

The meta-analysis pooled results are presented in Fig 4. The heterogeneity test indicated that a fixed-effects model could be selected. Thus, in the gemcitabine vs BSC/observation group, the pooled HR was 0.91 (0.76–1.09, p = 0.314; I^2^ = 0.0%, p = 0.856); in the pemetrexed vs BSC±placebo group, the pooled HR was 0.75 (0.65–0.87, p = 0.000; I^2^ = 0.0%, p = 0.000); in the pemetrexed +bevacizumab vs bevacizumab group, the HR was 0.98 (0.85–1.12, p = 0.744; I^2^ = 0, p = 0.481), in the pemetrexed vs bevacizumab group, the HR was 1.03 (0.83–1.12, p = 0.763; I^2^ = 0, p = 0.580).

### Grade 3–4 AEs

**Fig 5:**
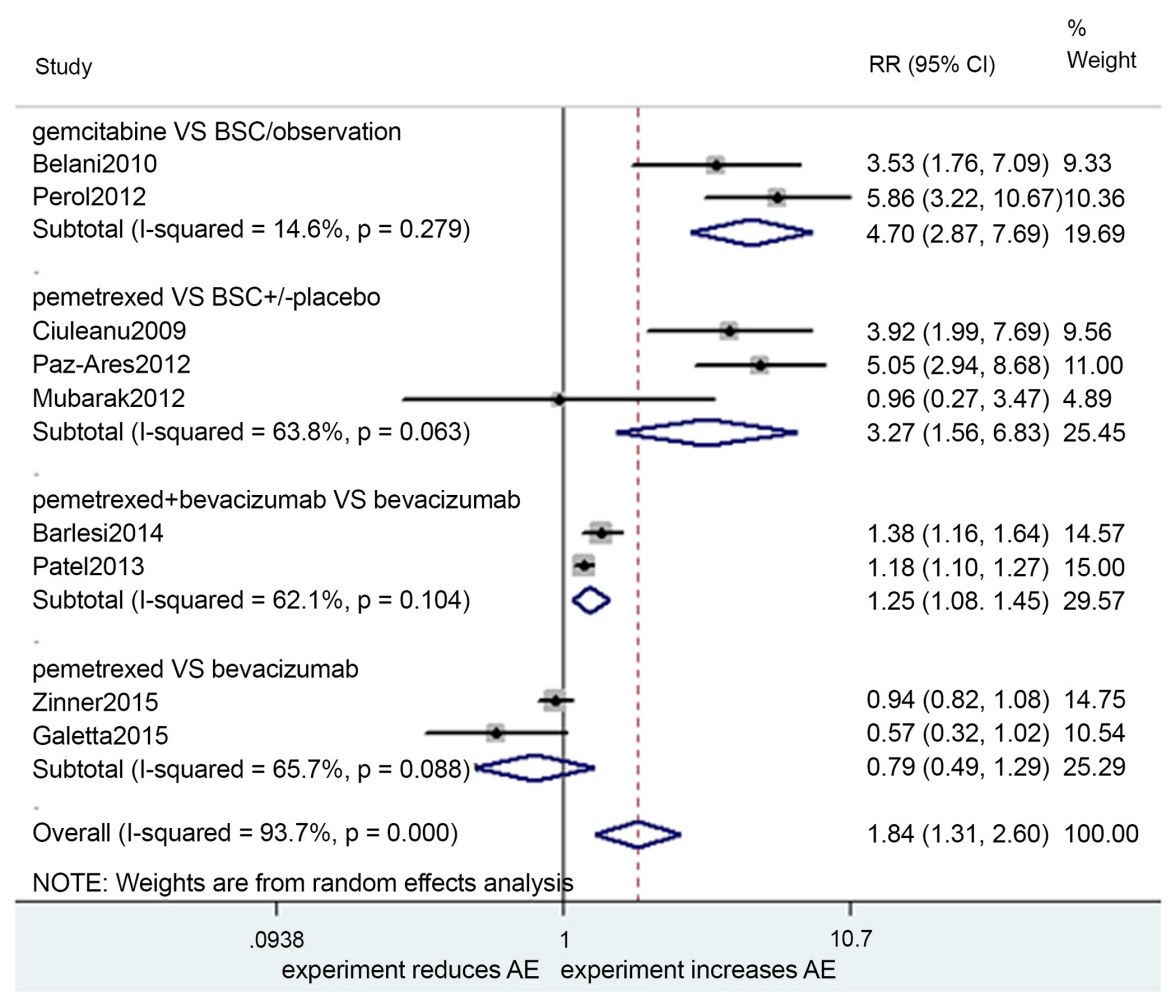
Meta-analysis results of grade 3–4 AEs.

The meta-analysis pooled results are presented in Fig 5. The heterogeneity test indicated that a random-effects model could be selected. Thus, in the gemcitabine vs. BSC/observation group, the pooled HR was 4.70 (2.87–7.69, p = 0.000; I^2^ = 14.6%, p = 0.279). In the pemetrexed vs. BSC ± placebo group, the pooled HR was 3.27 (1.56–6.83, p = 0.002; I^2^ = 63.8%, p = 0.063). In the pemetrexed + bevacizumab vs. bevacizumab group, the HR was 1.25 (1.08–1.45, p = 0.002; I^2^ = 62.1%, p = 0.104). In the pemetrexed vs. bevacizumab group, the HR was 0.79 (0.49–1.29, p = 0.343; I^2^ = 65.7, p = 0.088).

### Sensitivity analysis

**Fig 6:**
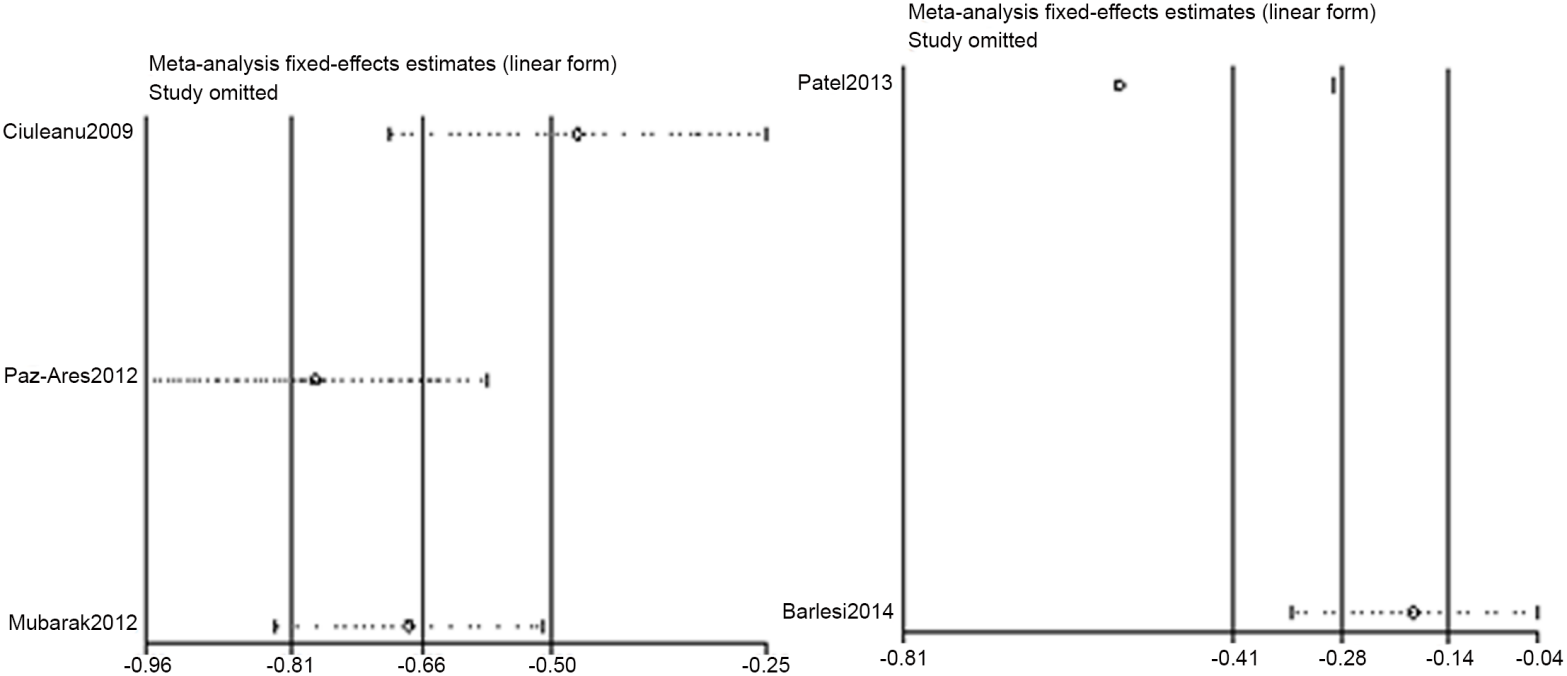
Sensitivity analysis result of PFS.

**Fig 7:**
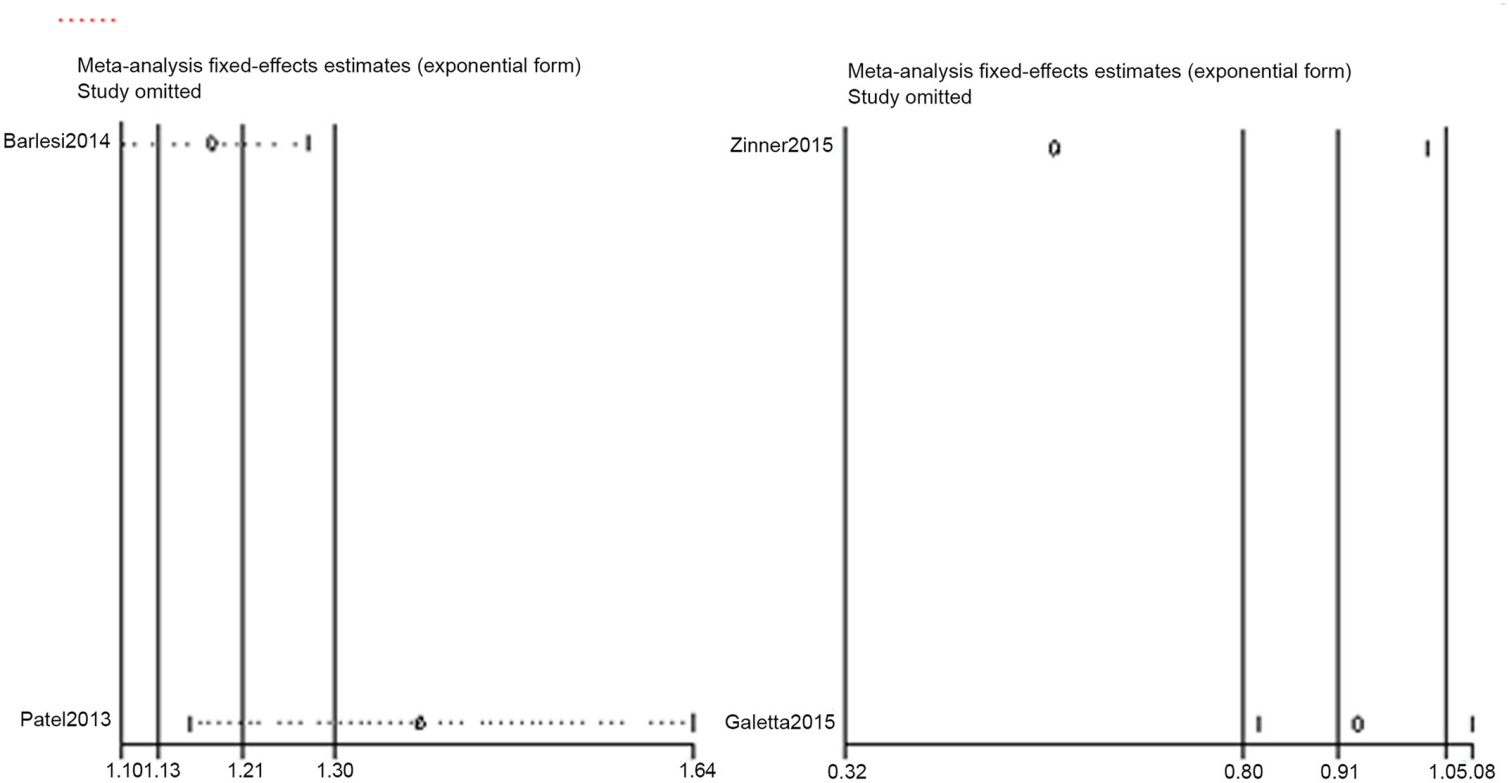
Sensitivity analysis result of grade 3–4 AEs.

Sensitivity analyses were conducted on PFS and grade 3–4 AEs to assess the heterogeneity. Thus, the PFS in the Ciuleanu and Patel studies and the grade 3–4 AEs in the Patel and Zinner studies likely contributed to the heterogeneity (Figs 6 and 7).

### Publication bias

Funnel plots and Egger's test were used to explore the publication bias if the value of one study was ≥ 3. No evidence of significant publication bias was noted regarding PFS (pemetrexed vs. BSC: p = 0.699), OS (gemcitabine vs. BSC: p = 0.720; pemetrexed vs. BSC: p = 0.652) and AEs (pemetrexed vs. BSC: p = 0.388).

## Discussion

### Evidence Summary

#### Key findings and grades of evidence

In this meta-analysis, we separately conducted a meta-analysis for the gemcitabine vs. BSC/observation, pemetrexed vs. BSC±placebo, pemetrexed + bevacizumab vs. bevacizumab and pemetrexed vs. bevacizumab groups. We were careful to avoid mixing the groups. Instead of using previous quality assessment tables, we adopted the Cochrane-recommended Cochrane Collaboration’s risk of bias graph to assess the risk of bias and the GRADE system to assess the grading of evidence in the outcome of the meta-analysis to more objectively evaluate the bias risk and the evidence grading of studies. The Cochrane Collaboration’s risk of bias graph revealed that the overall bias of all of the included studies was moderate. Among these studies, two double-blind studies exhibited low bias. The GRADE system revealed that the overall grading of evidence in the gemcitabine vs. BSC/observation group was "MODERATE", and the pemetrexed VS BSC ± placebo group exhibited a "HIGH" rating. The pemetrexed + bevacizumab vs. bevacizumab group and pemetrexed vs bevacizumab group exhibited "LOW" grades.

In the gemcitabine vs. BSC/observation group, gemcitabine significantly improved PFS (HR = 0.62, p = 0.000, I^2^ = 0.0%) but did not significantly improve OS (HR = 0.91, p = 0.314, I^2^ = 0.0%). The grades of evidence of PFS and OS in the GRADE system were "MODERATE" and were attributed to the studies of Brodowicz [6]and Belani [14] (these studies did not describe whether they were open-label or double-blind) and the Perol study [7] (this was an open-label trial). Thus, all of the three studies displayed bias in allocation concealment and blinding performance. Regarding histology, all of three studies were NSCLC, include adenocarcinoma, squamous cell carcinoma, large cell carcinoma and other type. In subgroup analysis of the Perol [7] study, different benefits were not noted between the squamous and non-squamous sub-types, all of the remaining subgroups exhibited a benefit in PFS, but the benefit was more obvious in patients who had an objective response to induction treatment. The Brodowicz [6] and Belani [14] studies did not conduct a subgroup analysis. As for performance status (PS), in Belani’s study[14], only 36% of patients had a Eastern Cooperative Oncology Group (ECOG)≤1 at the time of randomization, but this data in Perol’s study [7] was 94.5% (292/309), and in Brodowicz’s study [6] 48.1% (99/206) of patients had a Karnofsky performance status (KPS)>80 scores. Regarding the grade 3–4 AEs, gemcitabine therapy significantly increased the grade 3–4 AEs (HR = 4.7, p = 0.000), and the effect was distinct. Only one study showed an RR > 2, and another study showed an RR > 5, thus increasing the scores of the evidence grade. Thus, the grade of evidence was "HIGH". The most common AE was neutropenia with an incidence of 13.3 to 20.8% [7, 14]. Our results were consistent with those of Zhang [13], in which the pooled HR of PFS was 0.53 (0.43–0.65) and that of OS was 0.88 (0.74–1.04). The Perol study was only published as an abstract.

In the pemetrexed vs. BSC±placebo group, pemetrexed improve both the PFS (HR = 0.54, p = 0.000; I^2^ = 59.8%, p = 0.083) and OS (HR = 0.75, p = 0.000, I^2^ = 0.0%). The grades of evidence for PFS and OS were both "HIGH", which were attributed to the two primary studies being double-blind trials with no bias in allocation concealment and blinding performance. Our sensitivity analysis revealed that the heterogeneity in PFS originated from the Ciuleanu [8] study. In that study, the HR of PFS for all NSCLC cases was 0.50 (0.42–0.61). When this HR was incorporated into this meta-analysis, the pooled HR was 0.55 (0.47–0.65, p = 0.000; I^2^ = 11%, p = 0.325). Additionally, regarding the squamous histology cases, the HR of PFS was 0.69 (0.49–0.98), and the HR of OS was 1.07 (0.77–1.50). In addition, the OS advantage disappeared. No additional subgroup data were available from the Ciuleanu [8] and Mubarak [26] studies, so we were unable to perform a subgroup meta-analysis to further assess the heterogeneity. In the Paz-Ares [9, 10] study, PFS and OS were improved in all of the subgroups. In patients with a complete response (CR) or partial response (PR), the HR in the CR or PR was 0.48. In patients with stable disease (SD), the HR was 0.74. Regarding grade 3–4 AEs, pemetrexed significantly increased the AEs (HR = 3.27, p = 0.002; I2 = 63.8%, p = 0.063). Because the sample size of the Mubarak [26] study was too small, after we excluded this study, the heterogeneity was absent (I^2^ = 0.0%), and the HR was 4.59. The evidence grade was also "HIGH" because one study had an RR > 2 and another study had an RR > 5, thus increasing the evidence grade scores. The most common grade 3–4 AEs were fatigue (5%), neutropenia (3–4%), anemia (3–4%) [8, 9,10].

In the pemetrexed + bevacizumab vs. bevacizumab group, the pemetrexed + bevacizumab group almost exhibited significantly improved PFS (HR = 0.71, p = 0.051; I^2^ = 81.5%, p = 0.020), but no obvious change in OS was noted (HR = 0.98, p = 0.744, I^2^ = 0.0%), thus significantly increasing the incidence of grade 3-4AEs (HR = 1.25, p = 0.002, I^2^ = 62.1%, p = 0.104). The evidence grade of PFS and grade of 3–4 AEs was "LOW", which could be attributed to the fact that all of the studies were open label trials and the large heterogeneity. However, regarding OS, the evidence grade was elevated to "MODERATE" because no heterogeneity was noted. The sensitivity analysis indicated that the heterogeneity in PFS and grade 3–4 AEs both originated from the Patel [20] study. This study lacked subgroup data to perform a subgroup meta-analysis, and which was limited in its design, which did not allow separate evaluation of the contribution of maintenance therapy to the efficacy outcomes.

In the pemetrexed vs. bevacizumab group, pemetrexed did not exhibit an obvious change in PFS (HR = 0.96, p = 0.752; I^2^ = 35.8%, p = 0.212) or OS (HR = 0.98, p = 0.744, I^2^ = 0.0%) but exhibited a slight trend to reduce grade 3–4 AEs (HR = 0.79, p = 0.343, I^2^ = 65.7, p = 0.088). The evidence grades of PFS and OS were "MODERATE". This result was attributed to the fact that these trials were open label, but large heterogeneity was not noted. However, regarding grade 3–4 AEs, the evidence grade decreased to "LOW" due to the obvious heterogeneity. The sensitivity analysis indicated that the heterogeneity was derived from the Zinner [27] study.

The Karayama [29] study assessed pemetrexed versus docetaxel in maintenance therapy after induction treatment with pemetrexed and carboplatin. The primary endpoint was survival without toxicity, and survival in the pemetrexed group (median: 20.8 months) was significantly increased compared with the docetaxel group (median: 0.5 months, HR = 0.36). However, the docetaxel group (8.2 months) exhibited an increased median PFS compared with the pemetrexed group (4.1 months), and the HR was 0.56 (p = 0.084). The OS in the pemetrexed group was increased (20.6 months) compared with the docetaxel group (19.9 months), and the HR was 0.79 (p = 0.622). Because this group only included one study, we did not use the GRADE system to assess the level of evidence.

#### Association with social economics

In recent years, the increasing emphasis on healthcare spending has placed growing pressure on policymakers. In the United States, US$2.8 trillion per year is spent on healthcare, a level that outpaced the gross domestic product (GDP) [30]. Several cost-effective studies of maintenance pemetrexed have been conducted according to the JMEN trial in the United States, United Kingdom, Switzerland, and Japan. The incremental cost-effectiveness ratio (ICER) per life year gained of maintenance pemetrexed was US$122,371 in the US (just below the accepted US standard of renal hemodialysis with an ICER of US$129, 090) [31]. An estimate of ICER was US$139,000 in Switzerland (above the nationally accepted willingness-to-pay threshold in Switzerland of €72, 000) [32], US$72,000 in the United Kingdom [33], and US$150,115 in Japan (above the Japanese threshold of US$43,478).

### Limitations

At the original study level: (1) The Belani [14] study was only an abstract, and the patient population had a worse PS at the time of randomization, which maybe induced a negative outcomes. (2)All of the open-label trials had a bias in allocation concealment and blinding performance. (3) The limitation in the designs of the Patel (2013) [20], Zinner (2015) [27] and Galetta (2015) [28] studies involves not separately evaluating the contribution of maintenance therapy to the efficacy outcomes. However, the other RCTs confirmed that the PFS or OS of pemetrexed was consistent in induction + maintenance therapy compared with maintenance therapy alone [9, 10].

2. At the systematic review and meta-analysis level: (1) We only searched the PubMed, Embase, Cochrane library, ASCO, ESMO, and NCCN databases and cannot account for other potentially relevant articles that were published in any other database. (2) Only a limited number of studies were included in the separate-group meta-analysis. (3) In the pemetrexed VS BSC ± placebo group, the Ciuleanu [8] study involved switch maintenance, whereas the Paz-Ares [9, 10] study involved continuation maintenance. Although they both revealed a change in PFS and OS, other differences remain unknown. (4) Sufficient subgroup data were not available to perform subgroup analysis to further explore heterogeneity.

## Conclusions

In our article, we confirmed that gemcitabine significantly improved PFS compared with BSC, pemetrexed significantly improved PFS and OS compared with BSC ± placebo, and pemetrexed + bevacizumab approached a significantly improved PFS compared with bevacizumab alone. The incidence of grade 3–4 AEs was significantly increased in the maintenance therapy arm compared with the control arm. Additional trials are required to confirm the impact of pemetrexed + bevacizumab vs. bevacizumab and pemetrexed vs. bevacizumab. In particular, randomized, controlled double-blind trials are required. Randomized, controlled double-blind trials are also needed for gemcitabine vs. BSC studies. In pemetrexed + bevacizumab vs. bevacizumab or pemetrexed vs. bevacizumab studies, the contribution of maintenance therapy to the outcomes should be separately evaluated. Finally, regarding the socioeconomic impact, the problems of maintenance therapy must identify new solutions.

## Supporting Information

S1 PRISMA Checklist PRISMA 2009 Checklist for this article. (DOC)

S1 Protocol Protocol for this systematic review. (DOC)
