# Supplementary material for: Role of Gemcitabine and Pemetrexed as Maintenance Therapy in Advanced NSCLC: A Systematic Review and Meta-Analysis of Randomized Controlled Trials
Source: PLoS One. 2016 Mar 8;11(3):e0149247. doi: 10.1371/journal.pone.0149247 (PMC4783083; doi:10.1371/journal.pone.0149247)

Perol2012，IFCT-GFPC0502 NCT00300586：


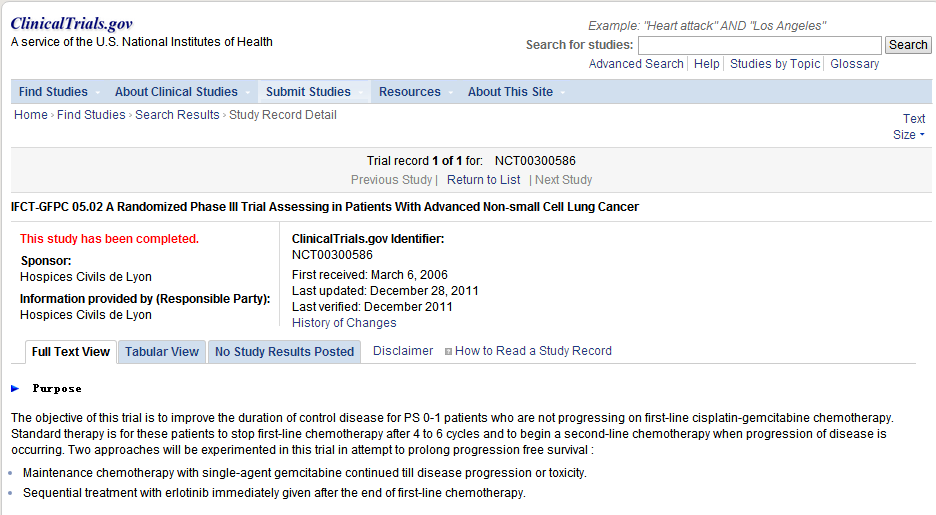

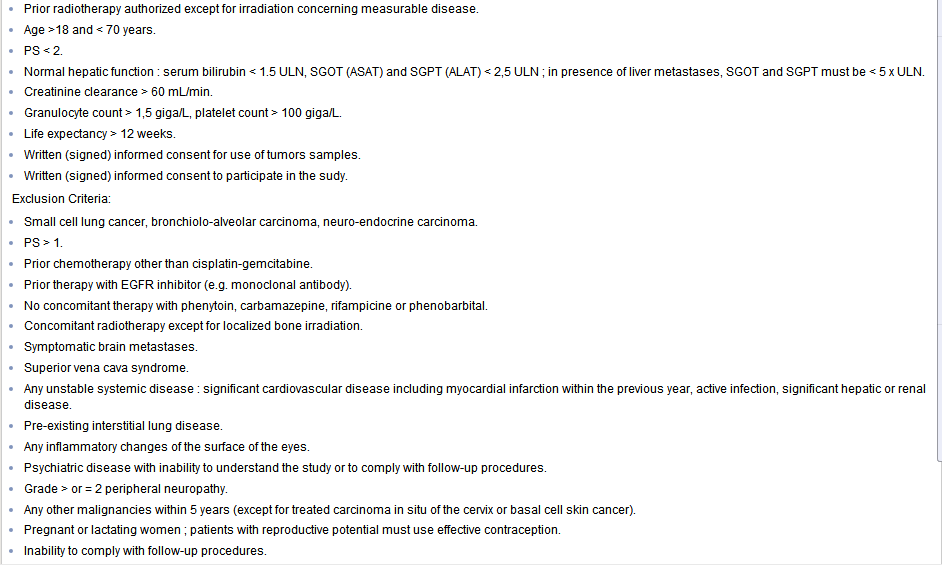

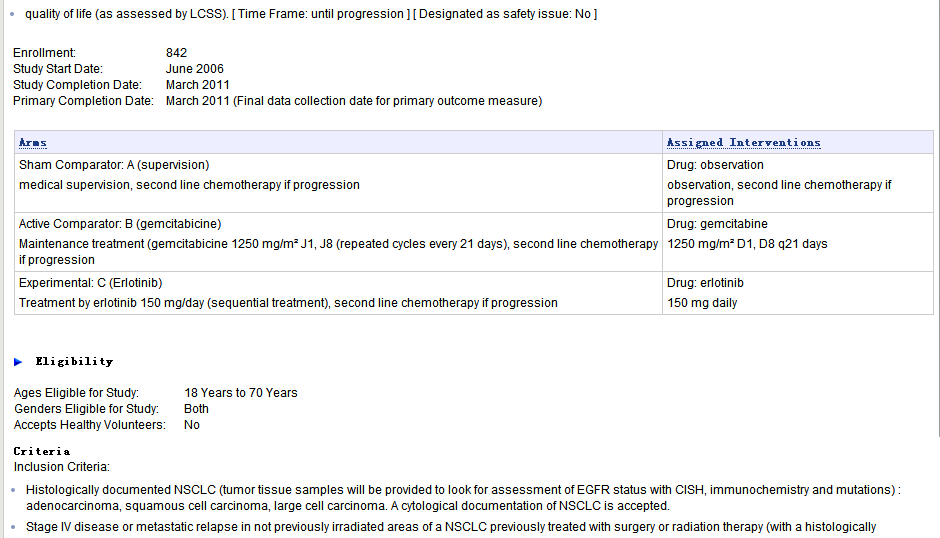

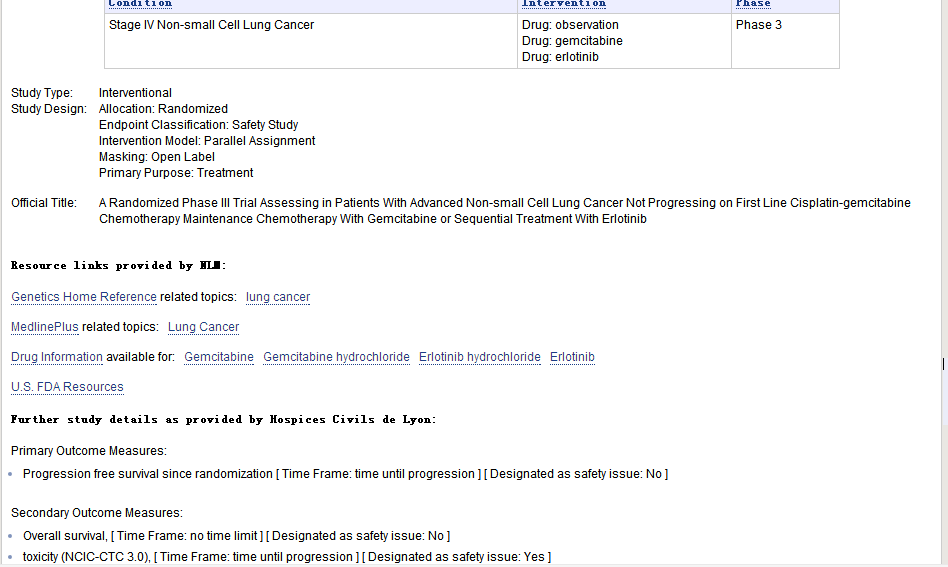

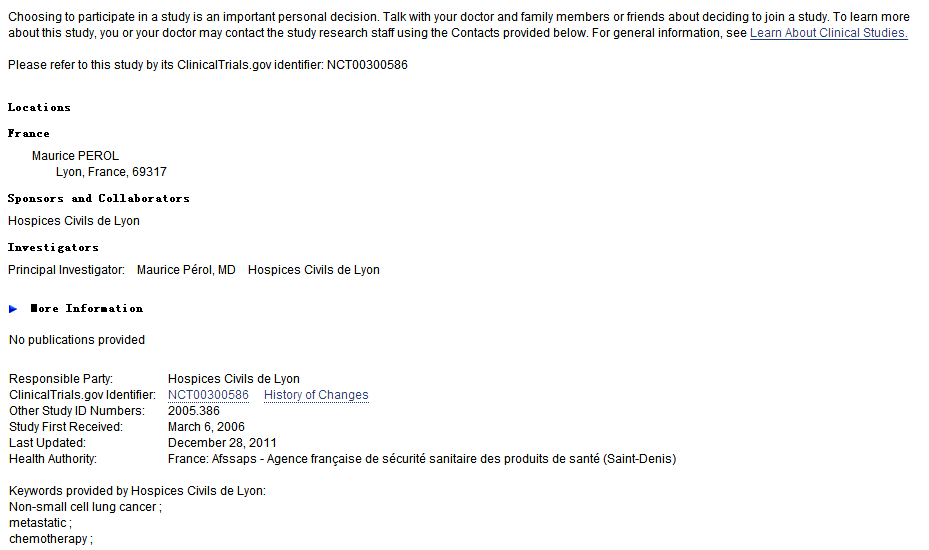


Ciuleanu2009，JMEN NCT00102804：


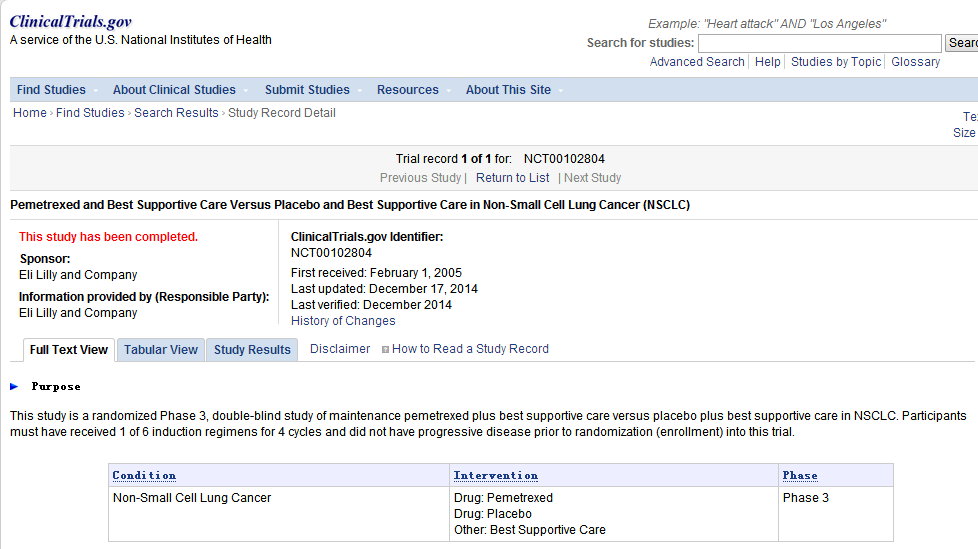


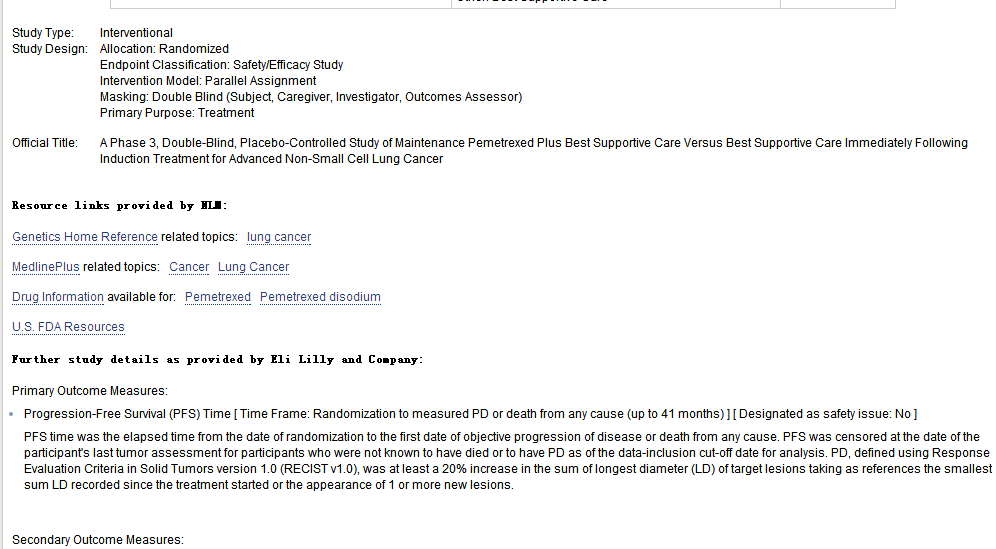


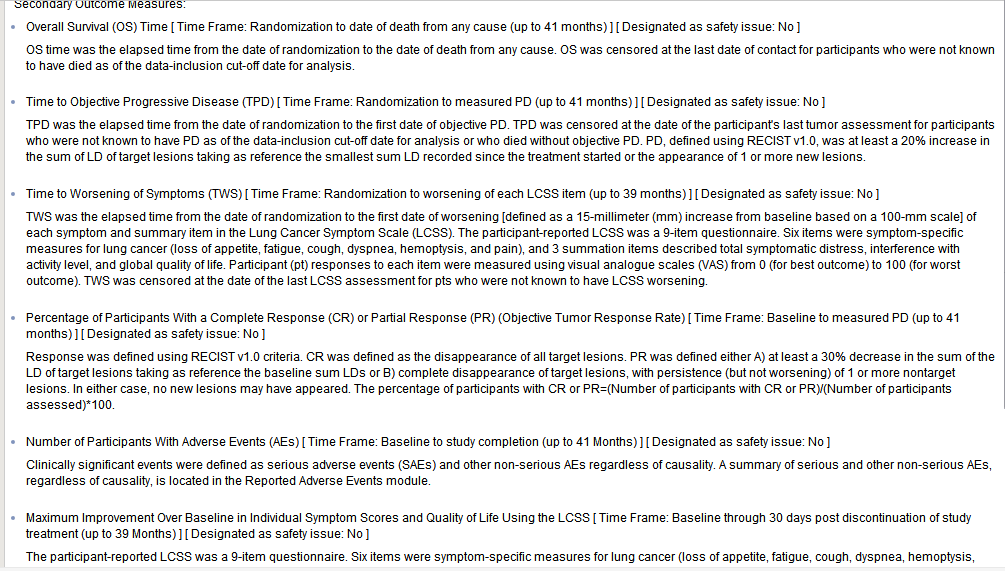

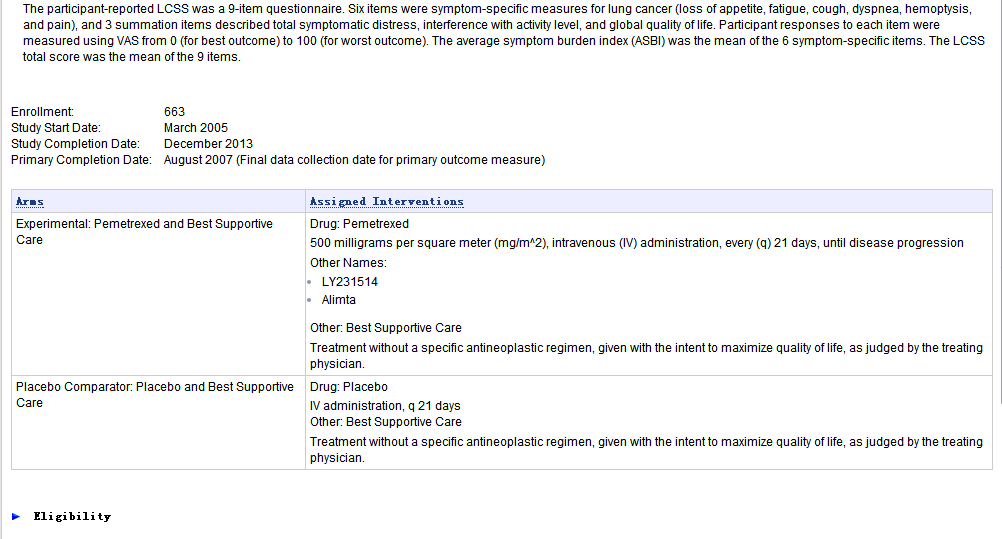


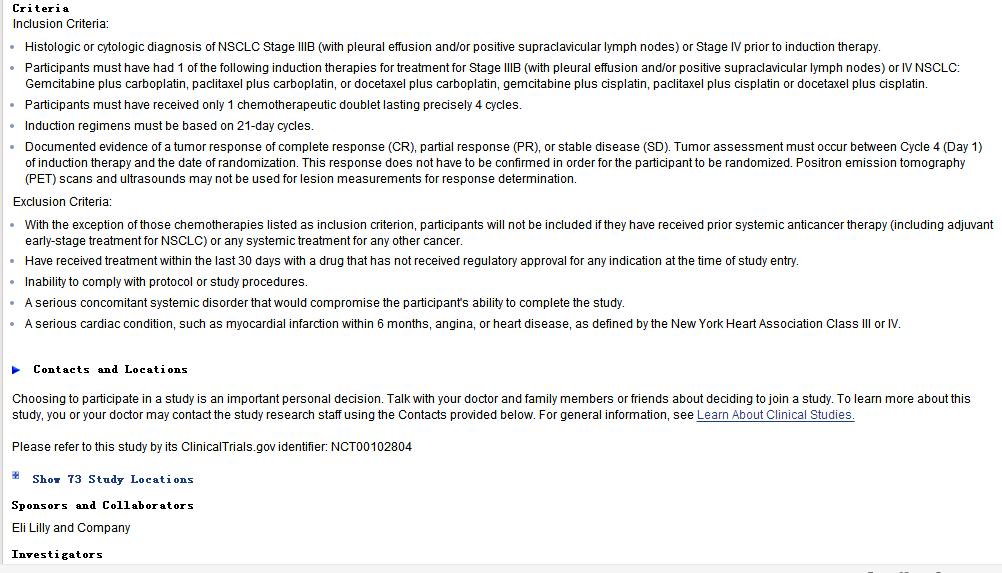


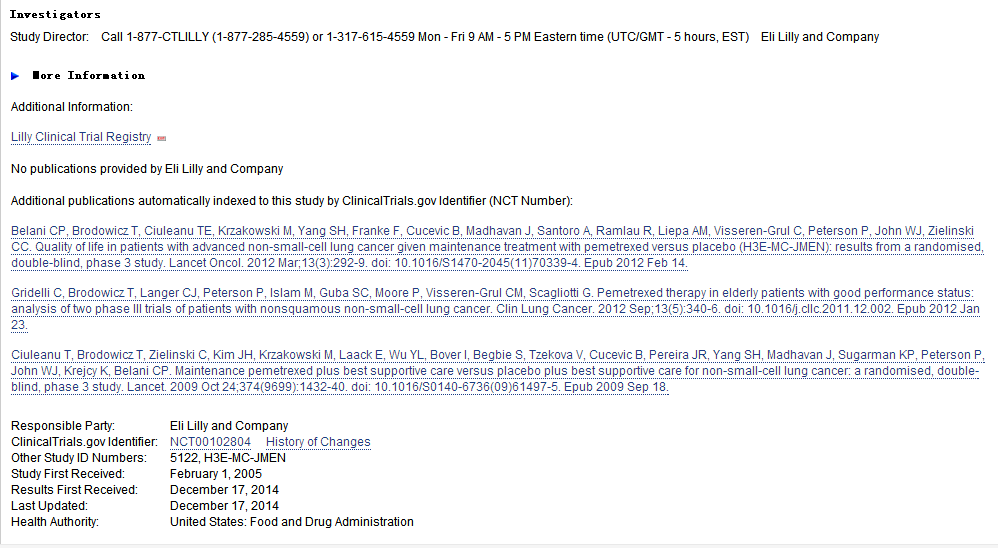


Paz-Ares2012 PARAMOUNT NCT00789373：


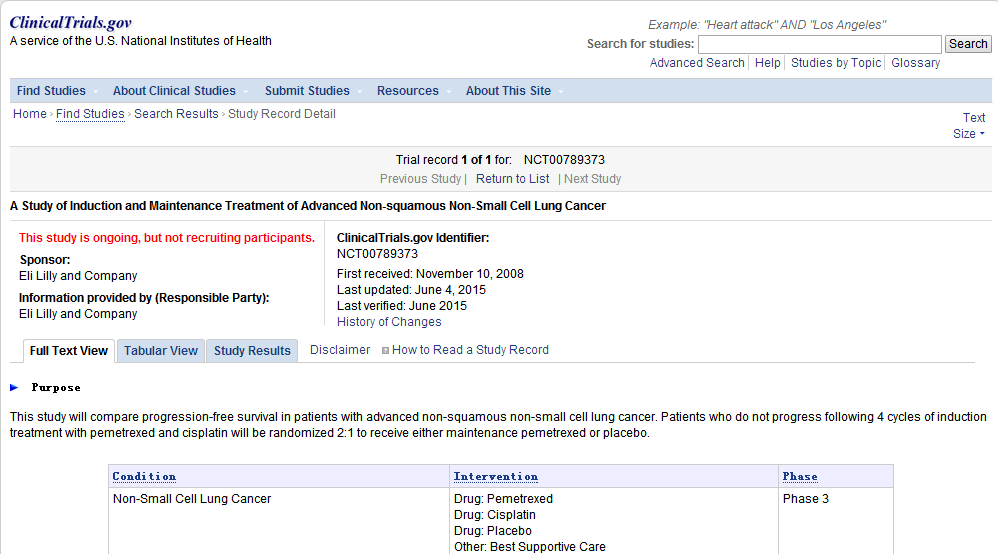

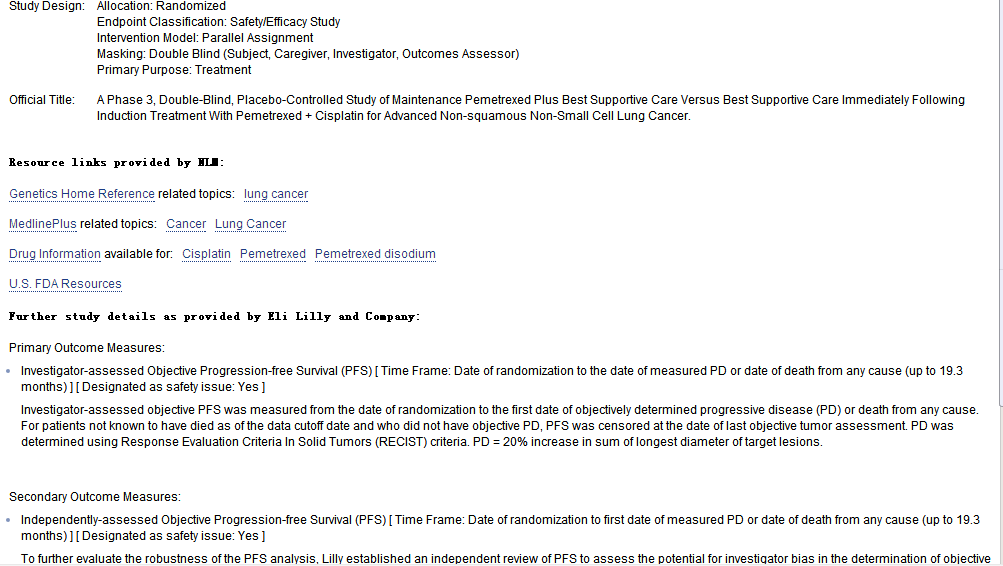

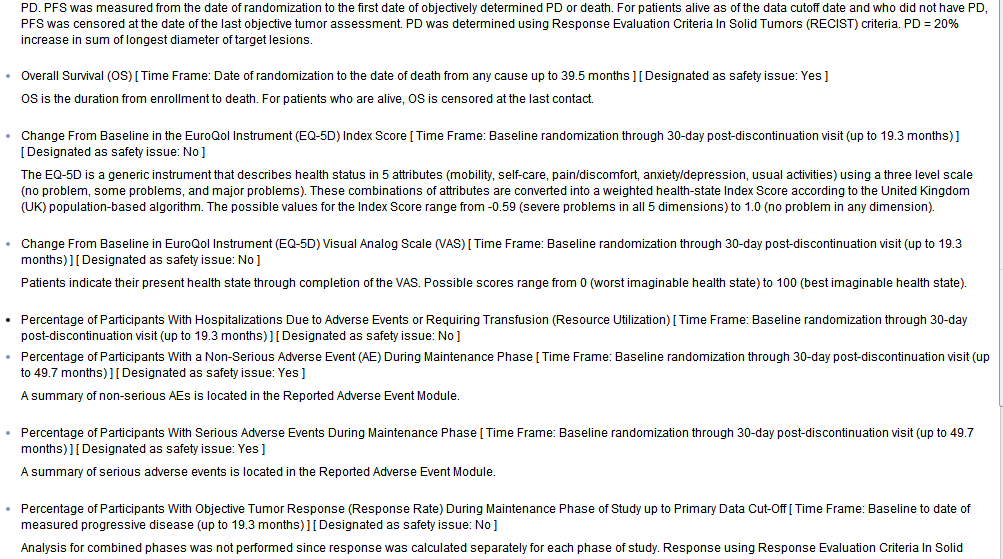

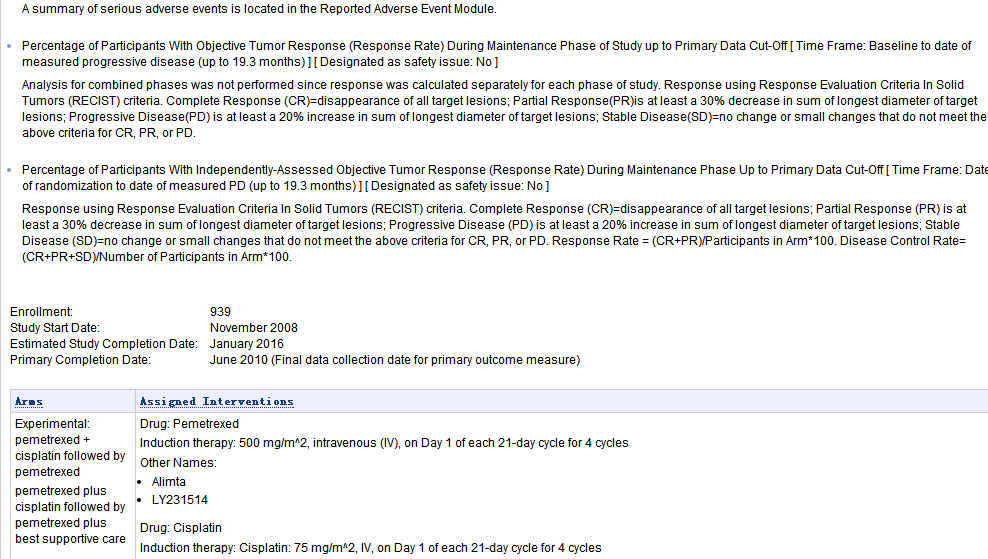

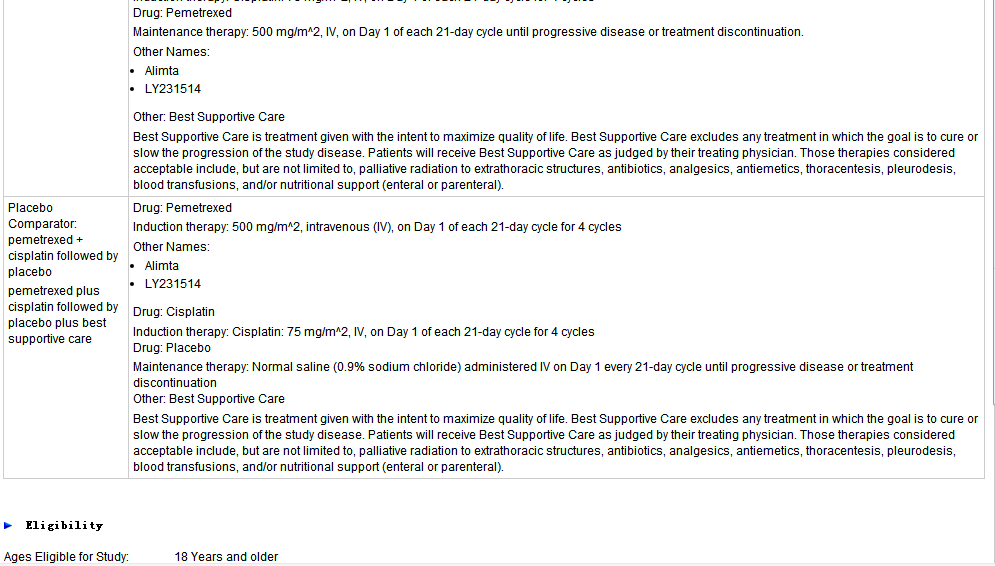

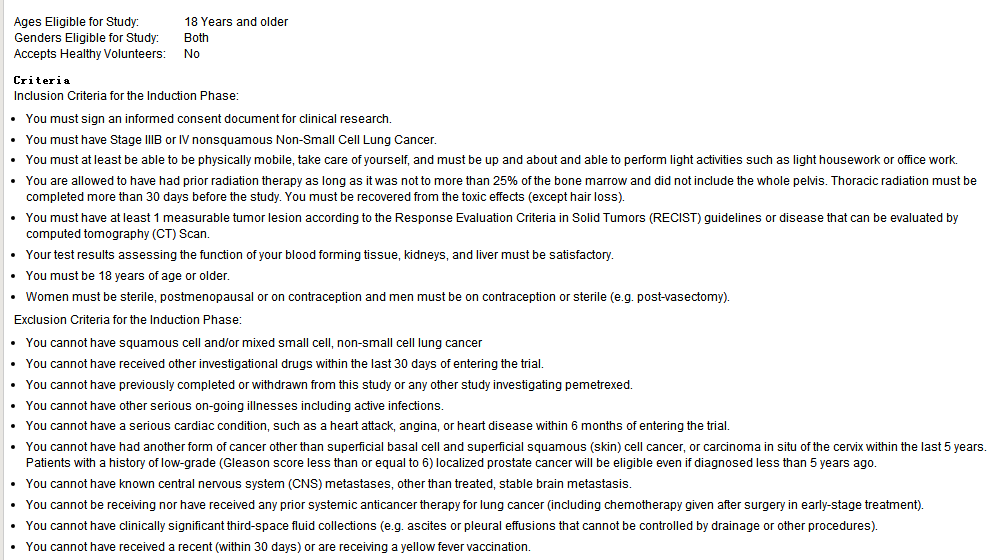

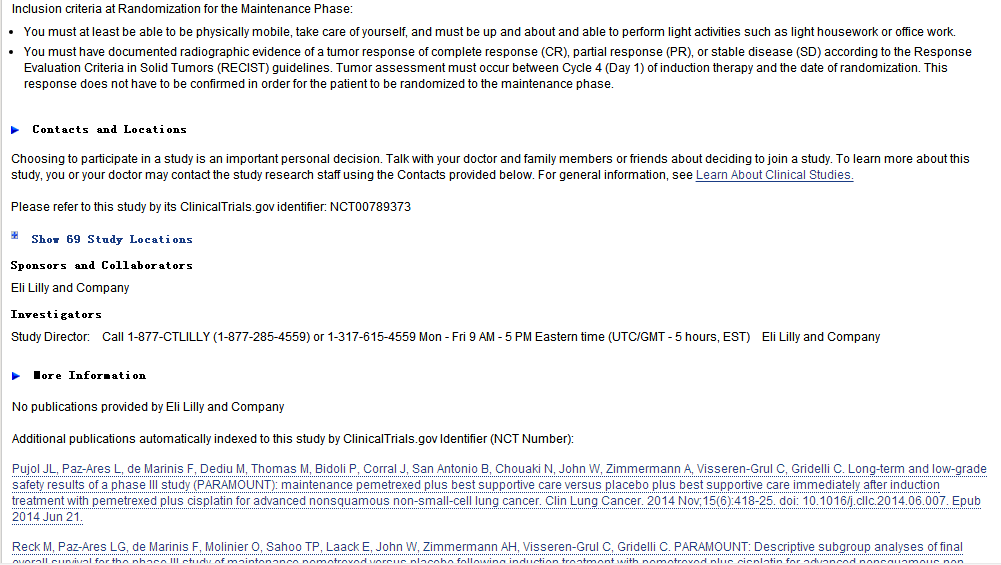

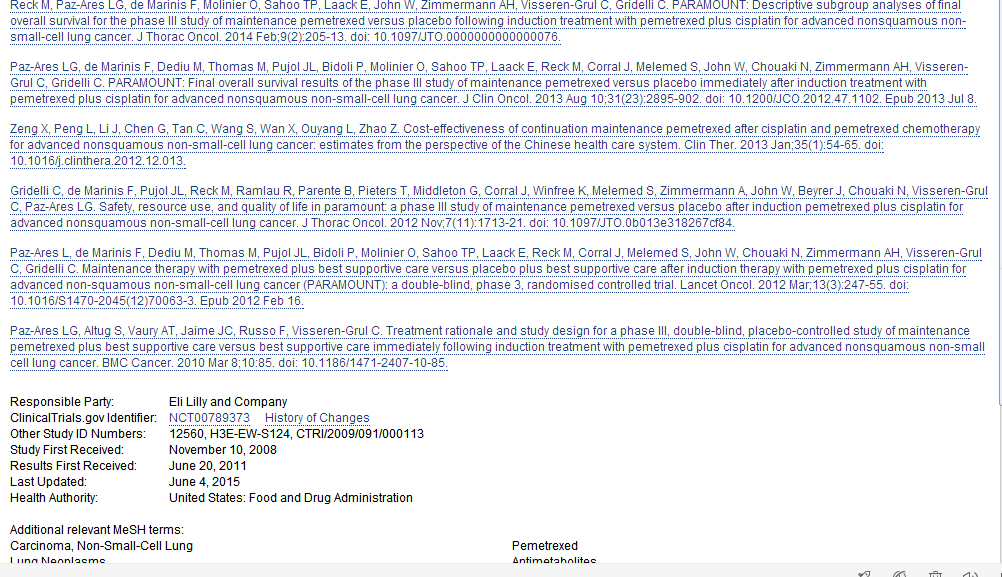


Mubarak2012 NCT00606021：


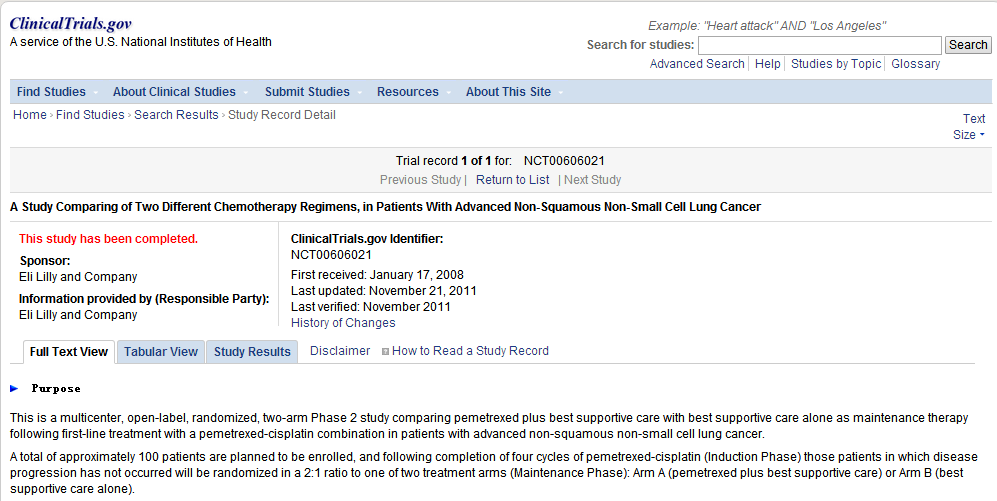

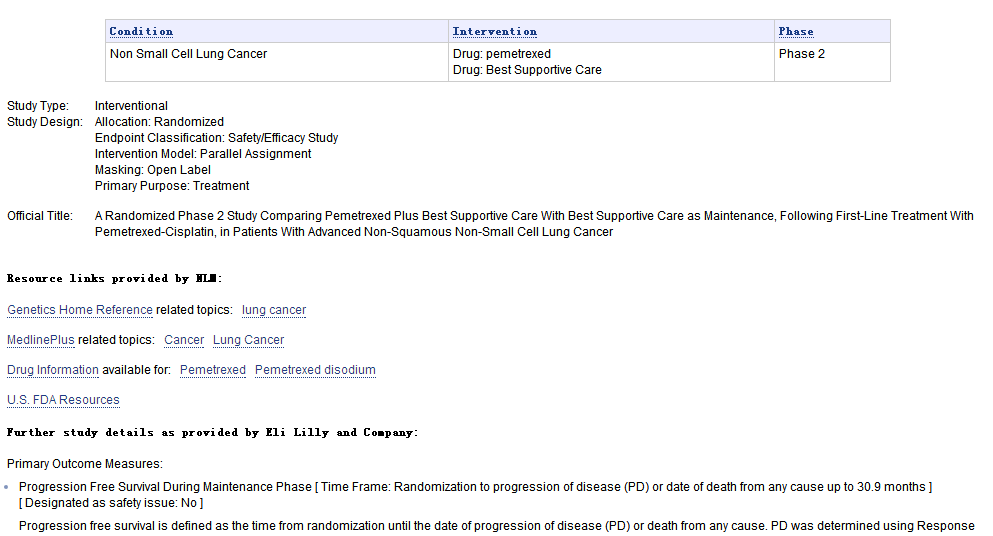


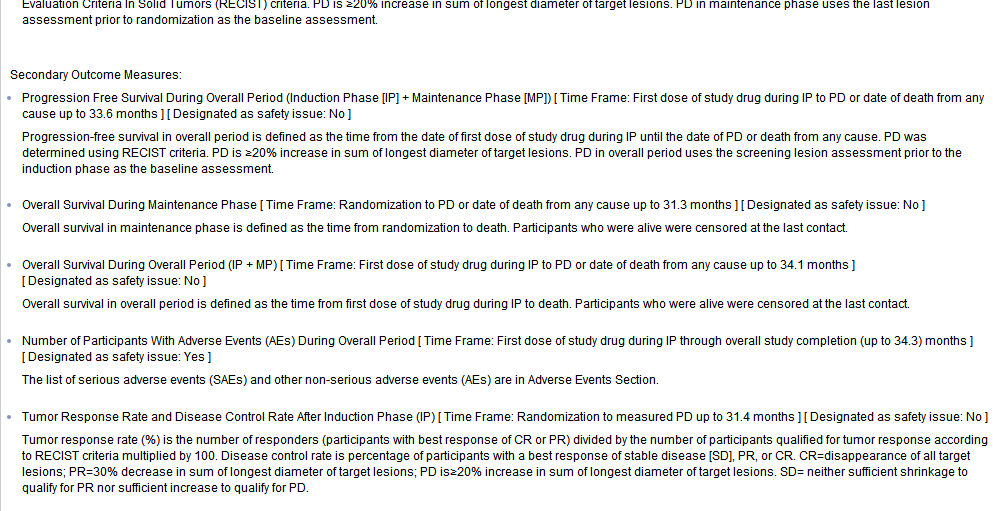

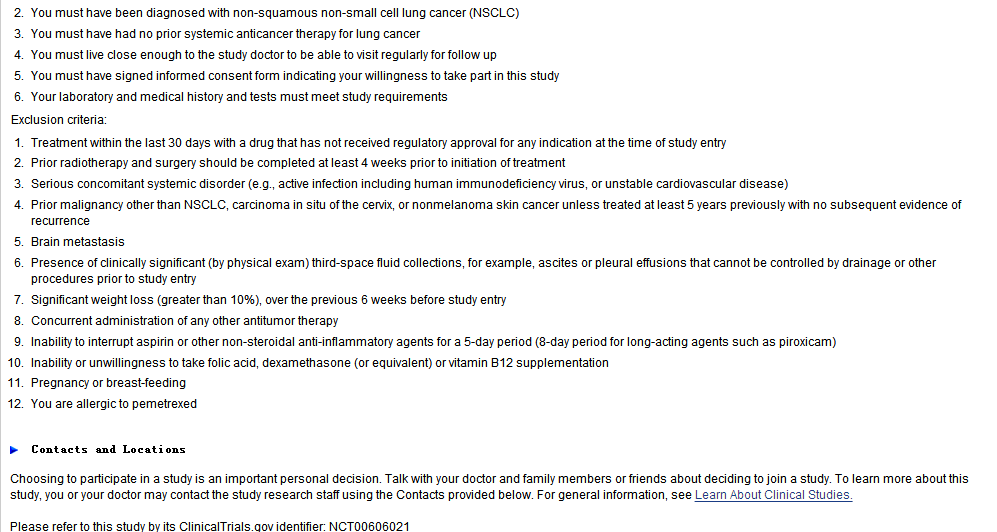

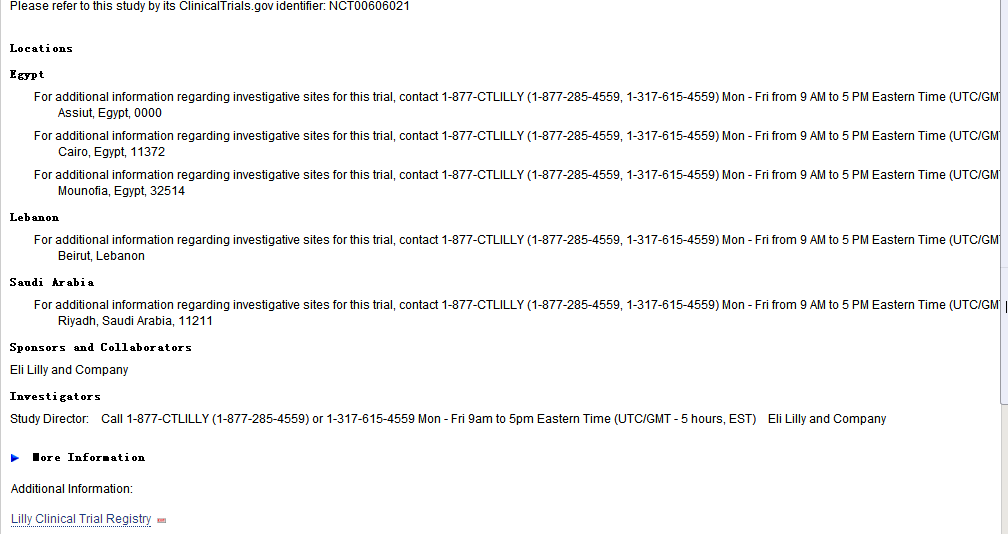


Barlesi2014 AVAPERL NCT00961415：


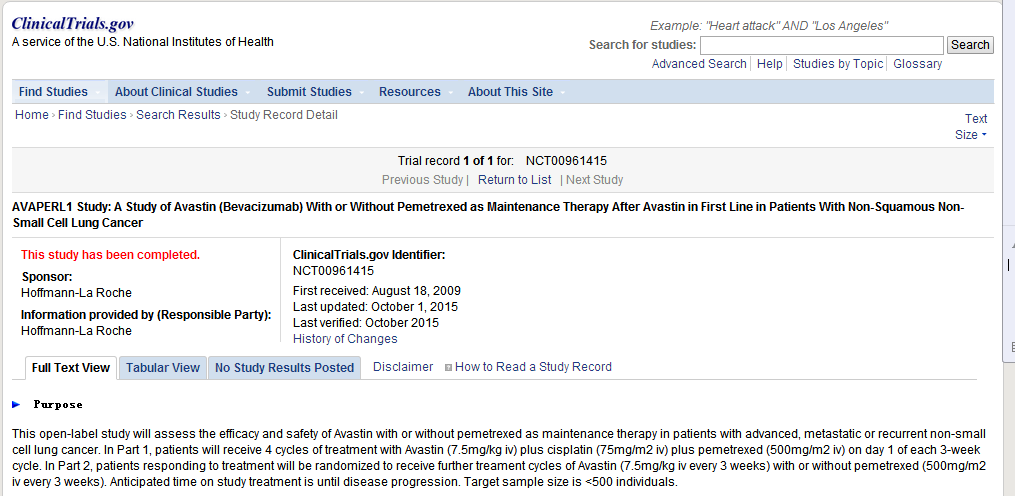

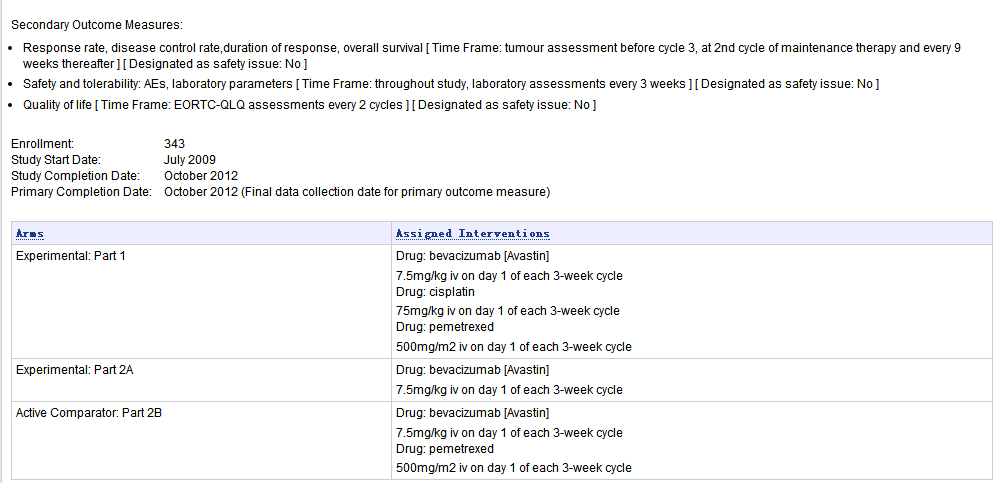

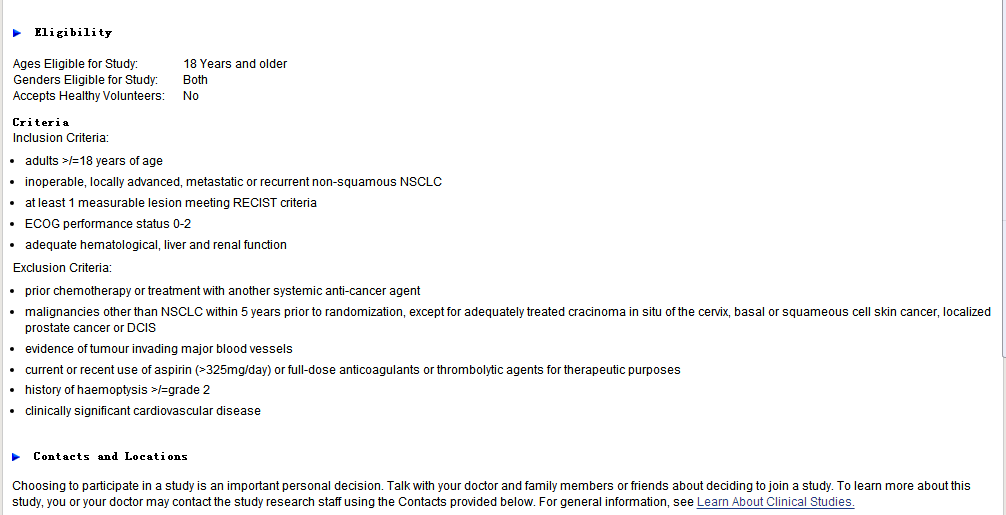

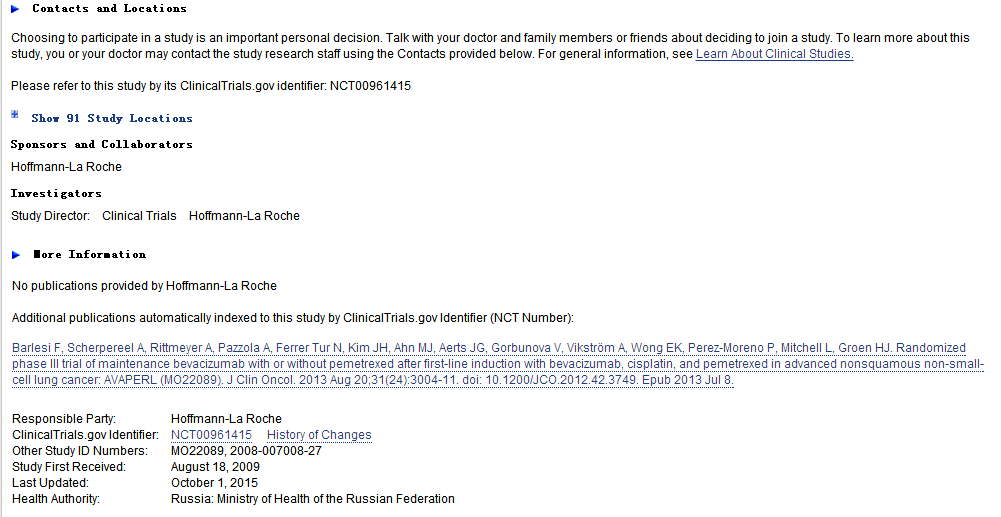


Patel2013 PointBreak NCT00762034：


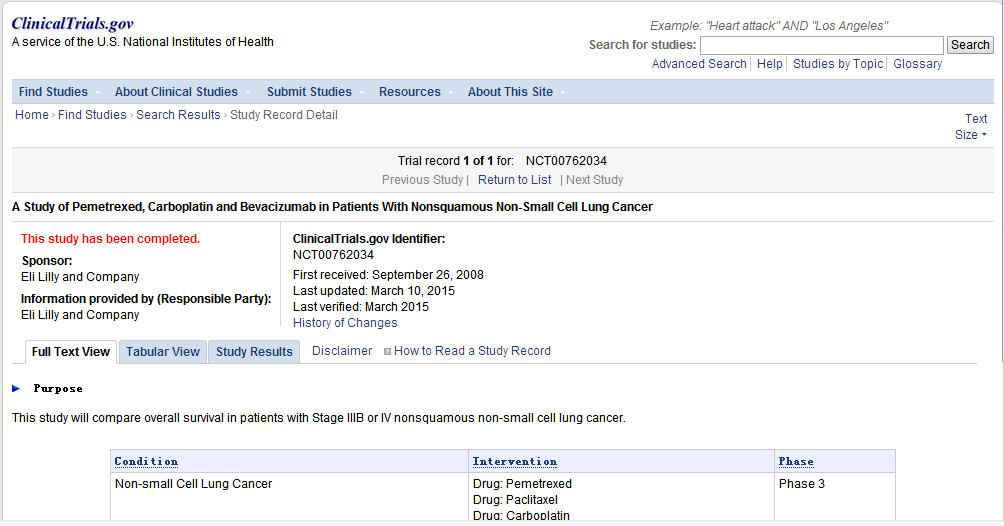


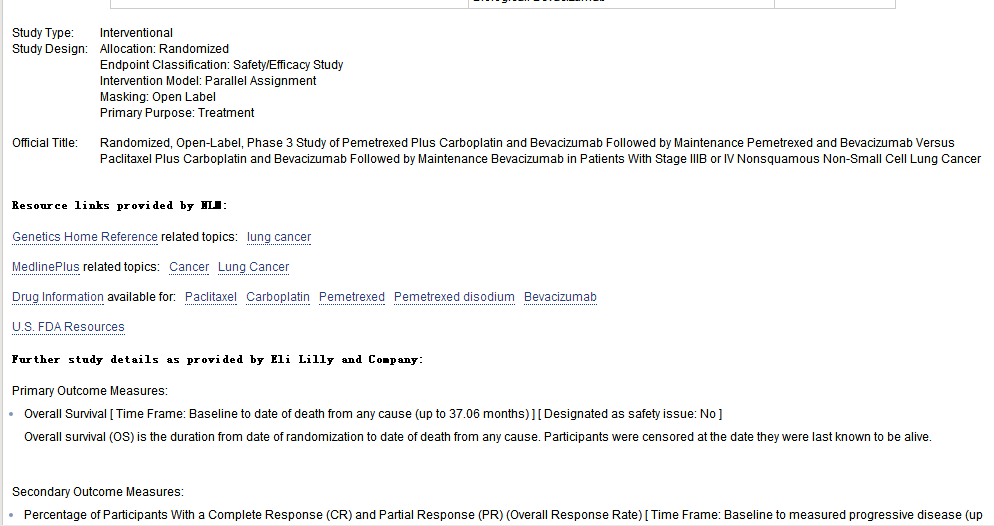

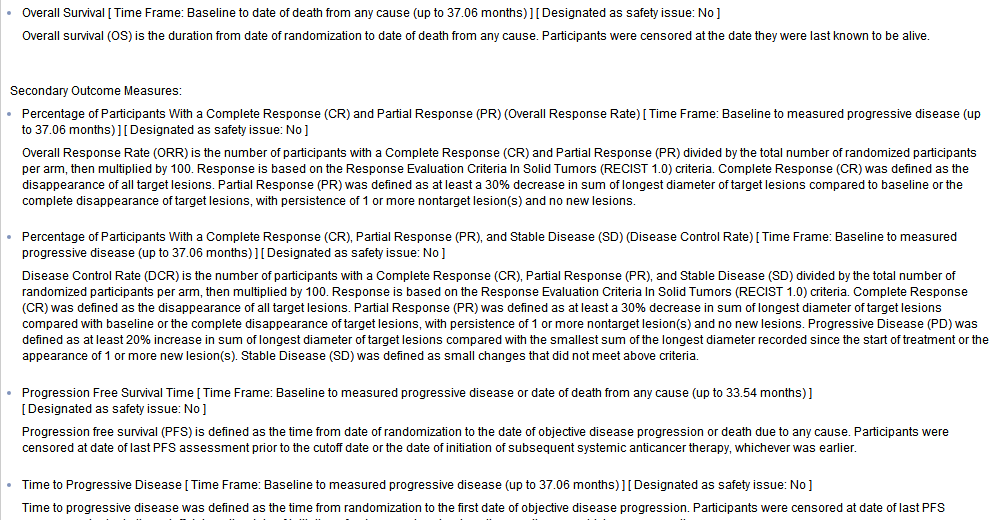

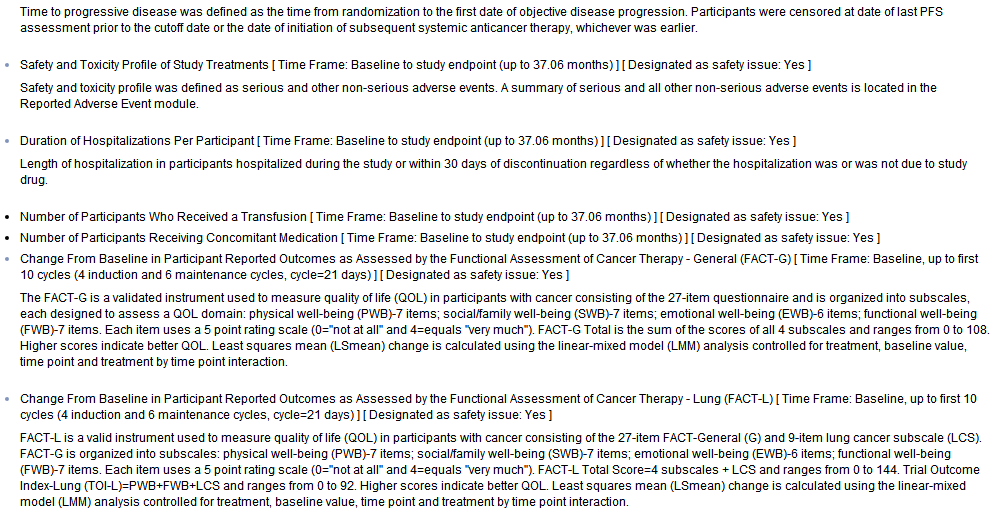

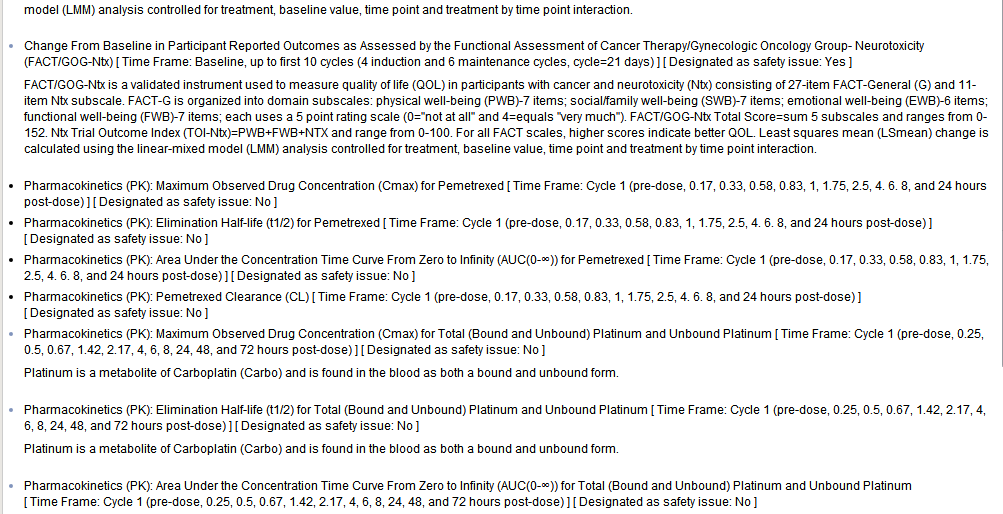

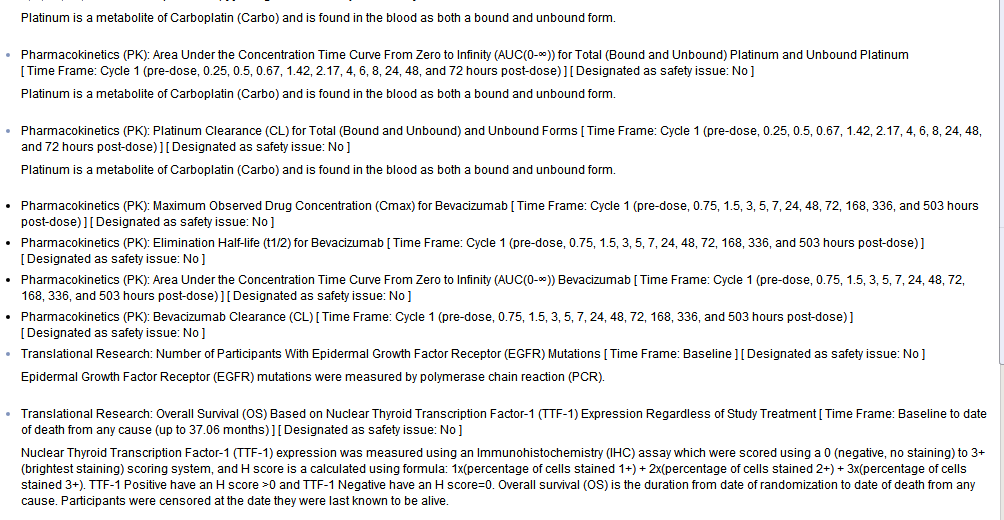

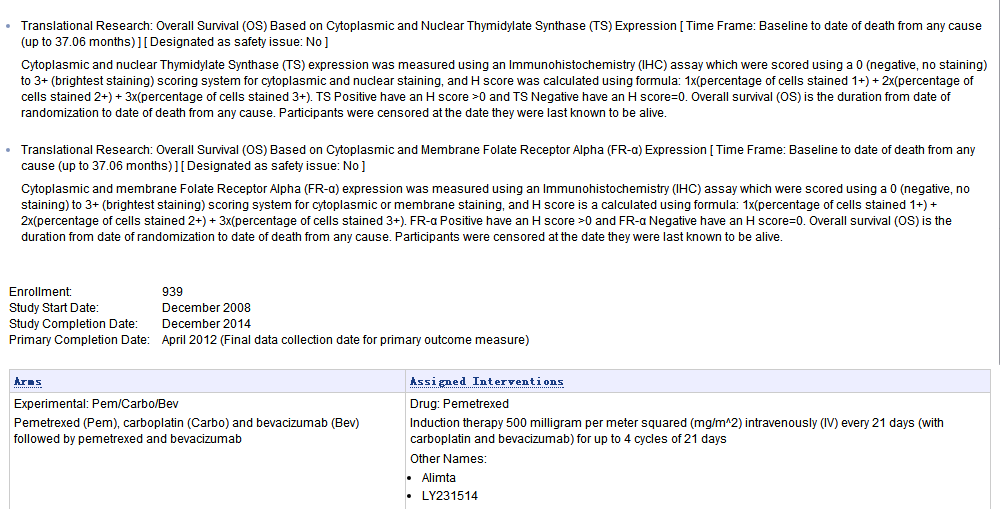

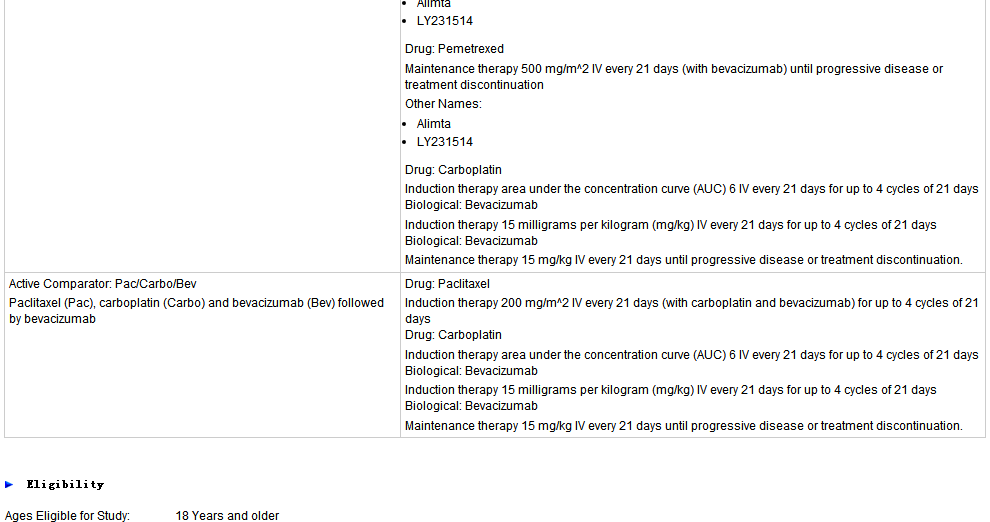

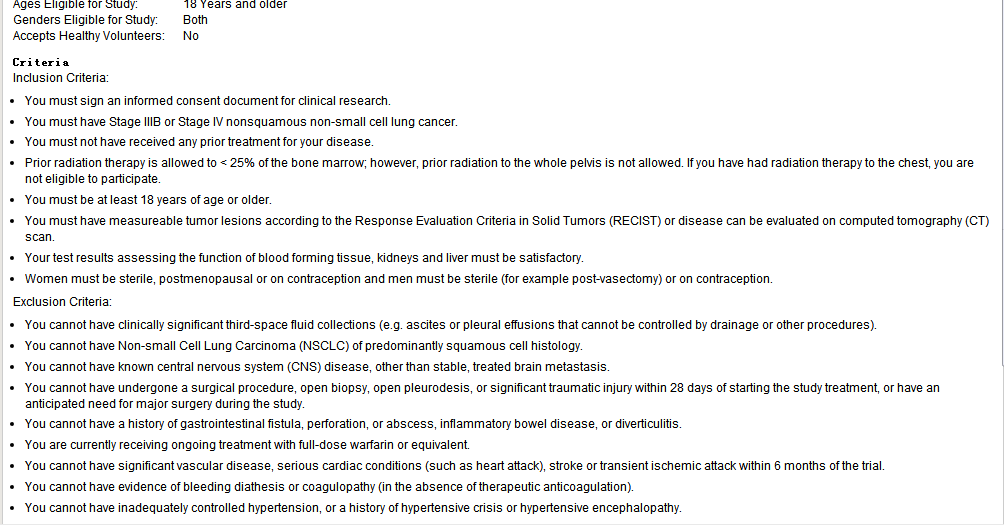

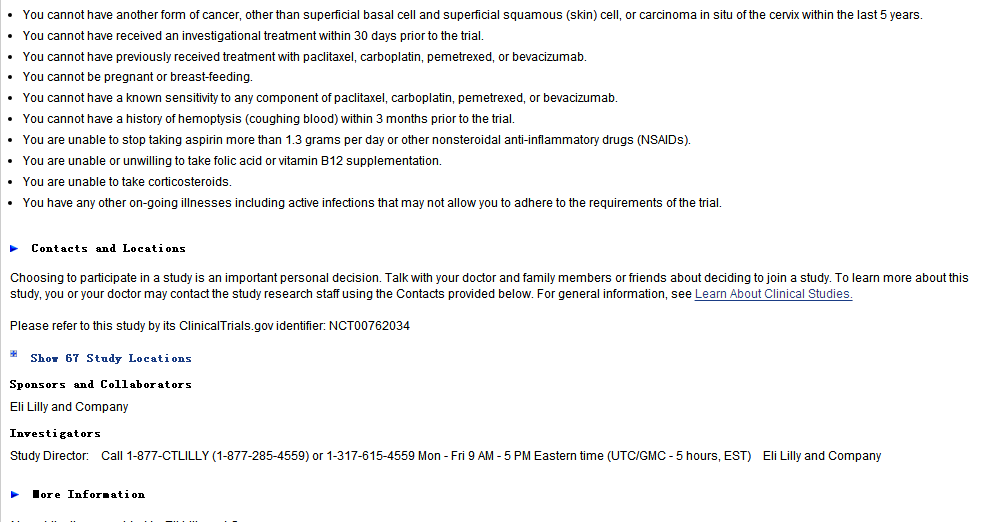

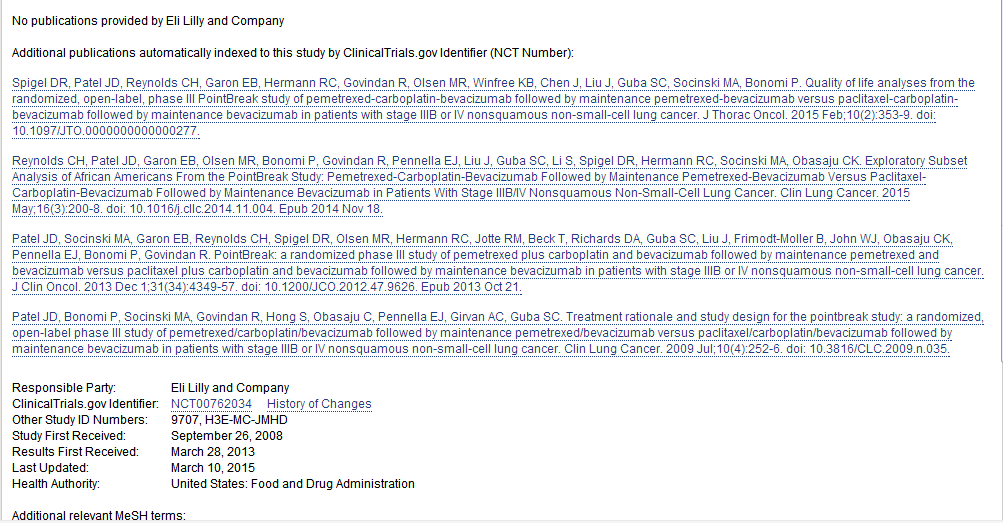


Zinner2015 PRONOUNCE NCT00948675：


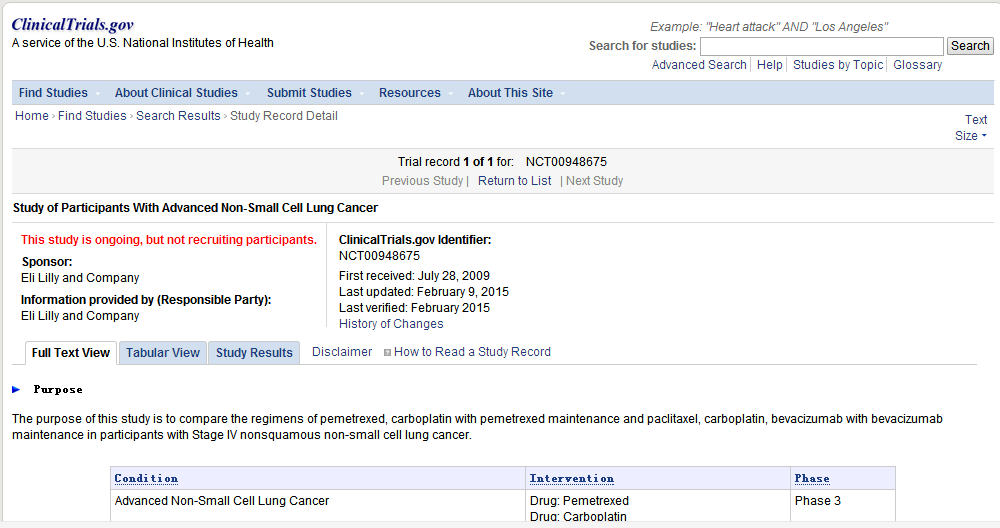

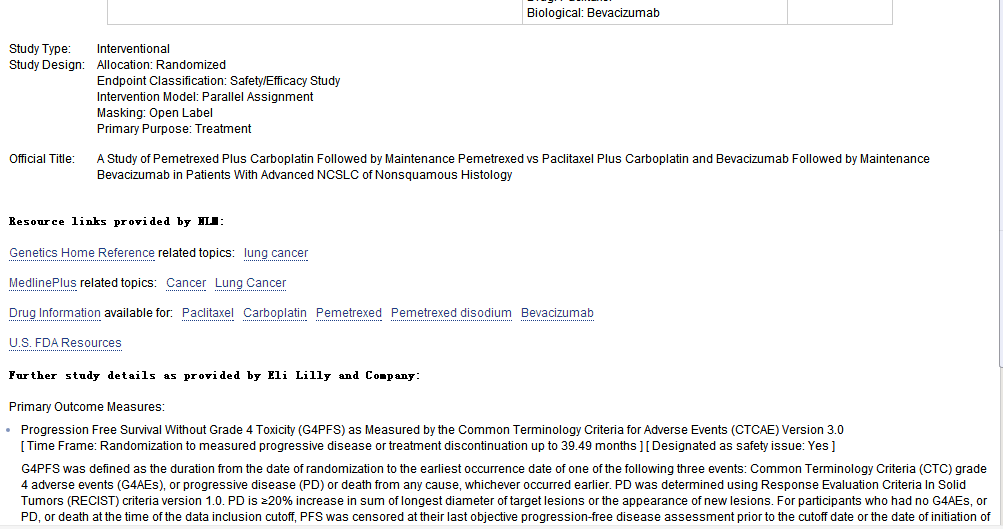

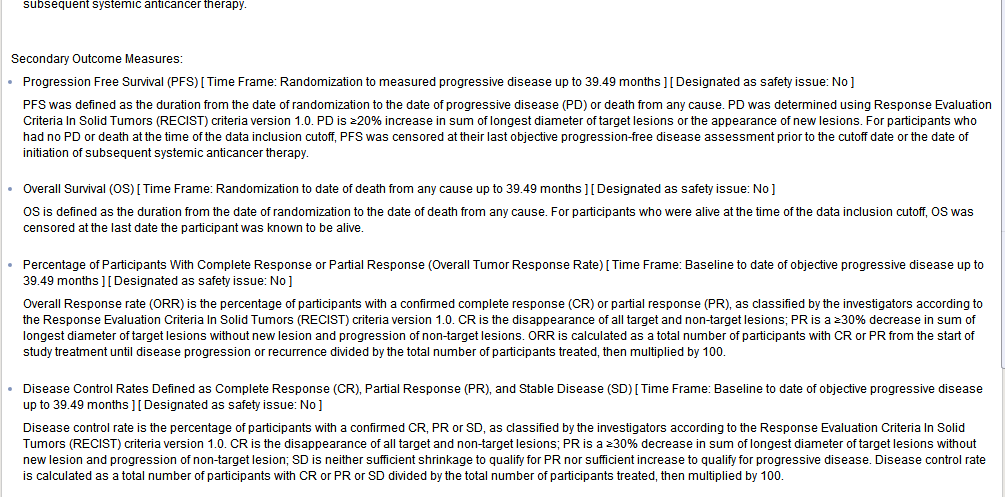

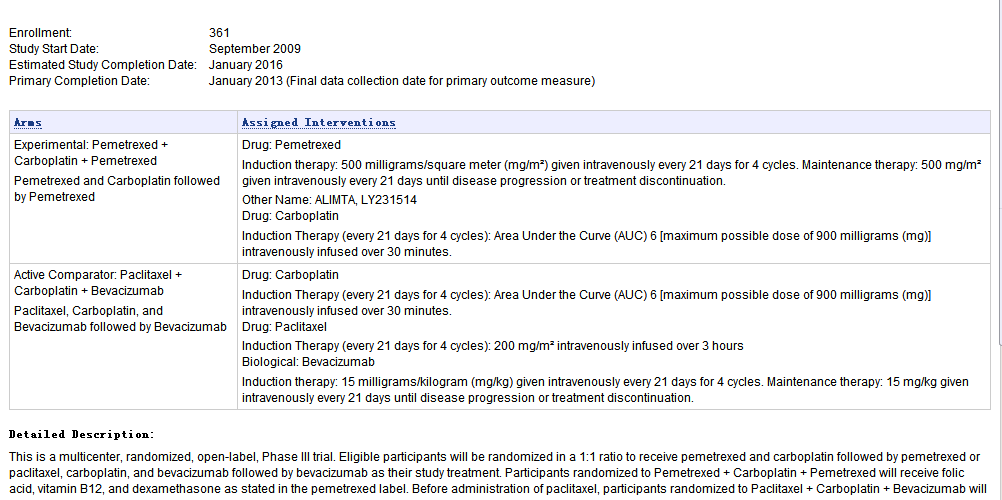

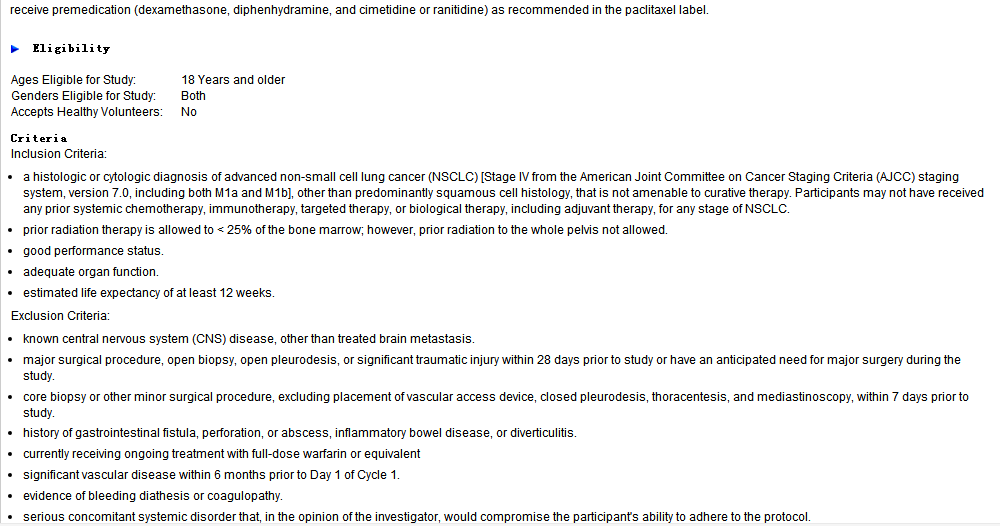

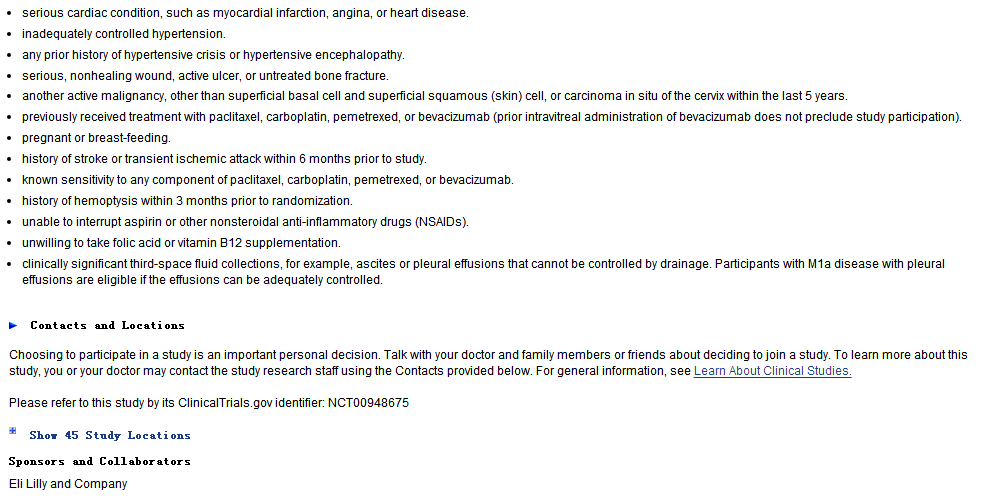

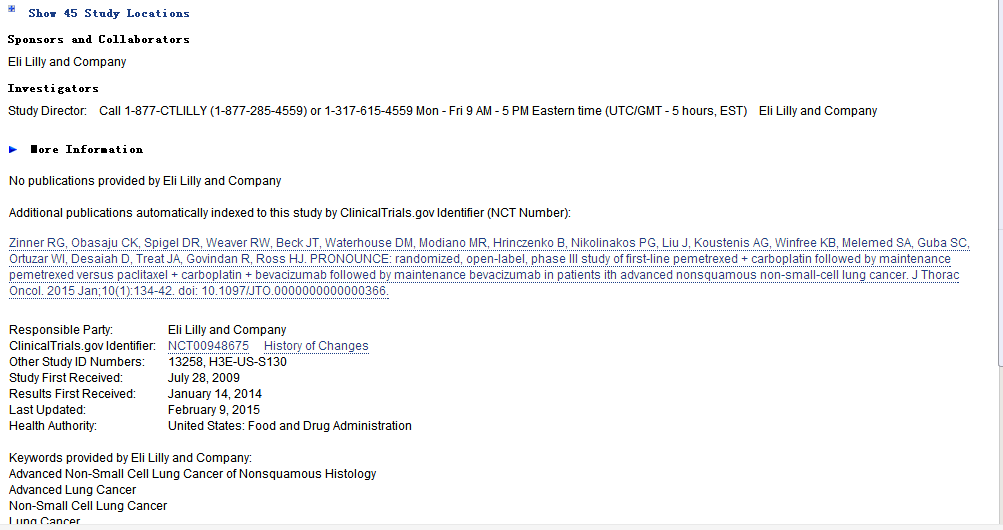


Galetta2015 ERACLE NCT01303926：


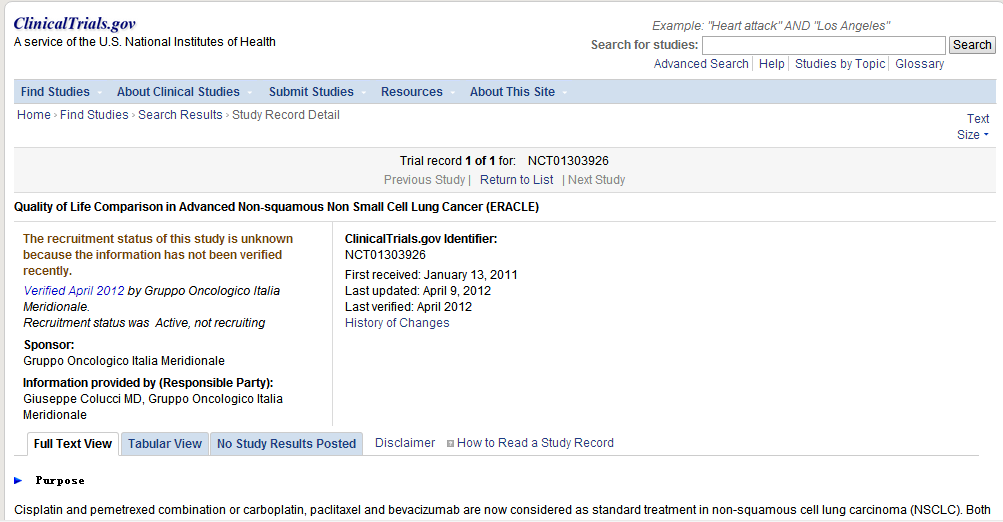

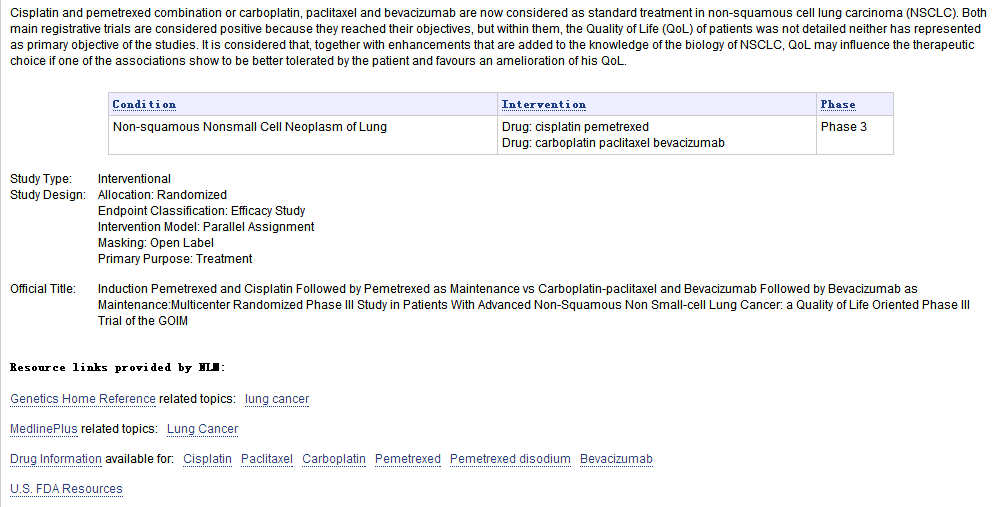

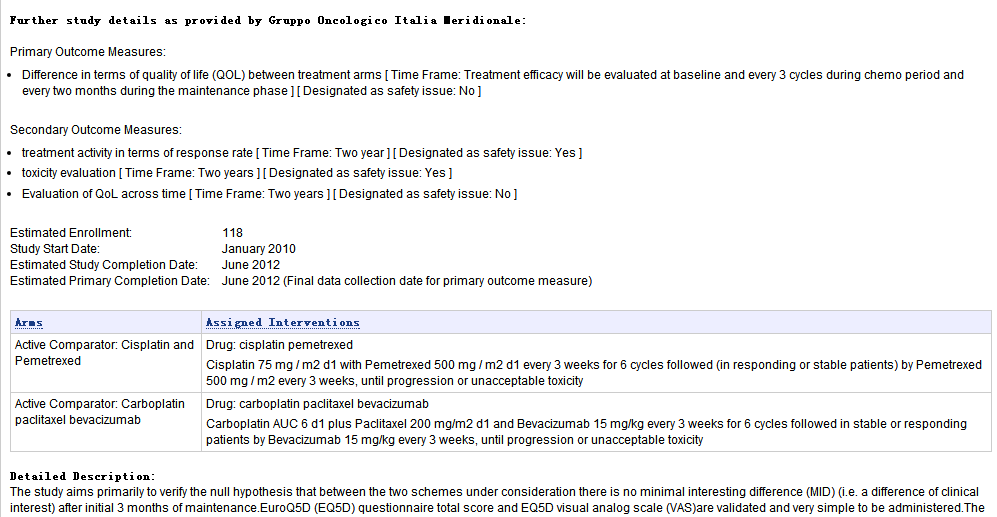

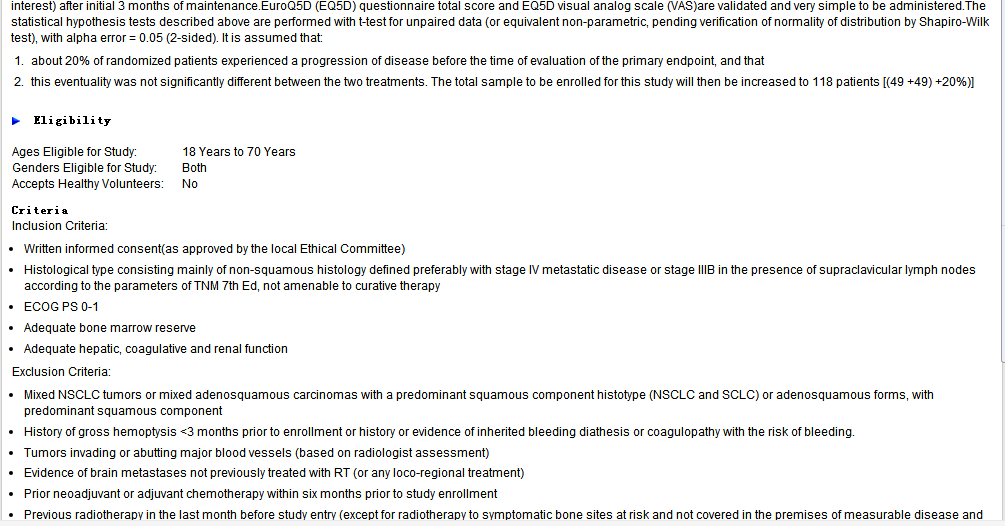

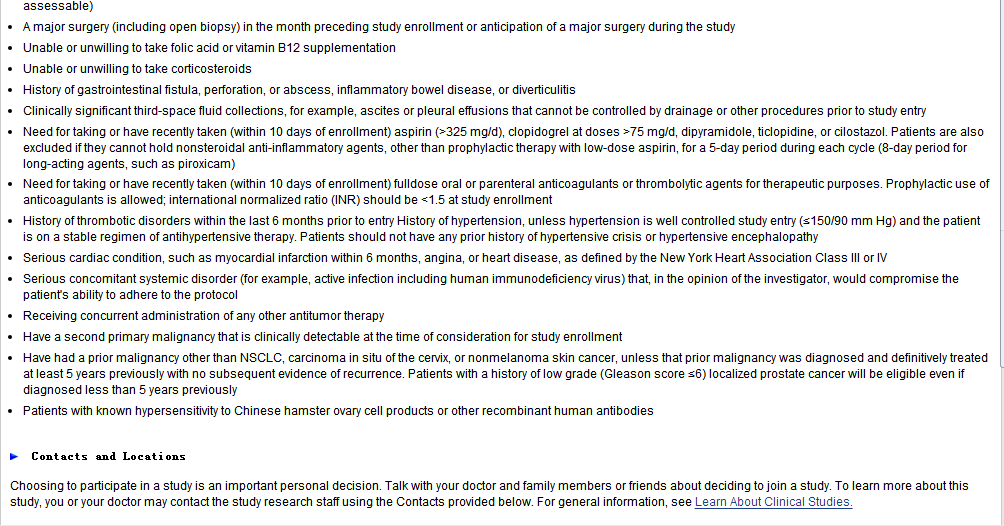

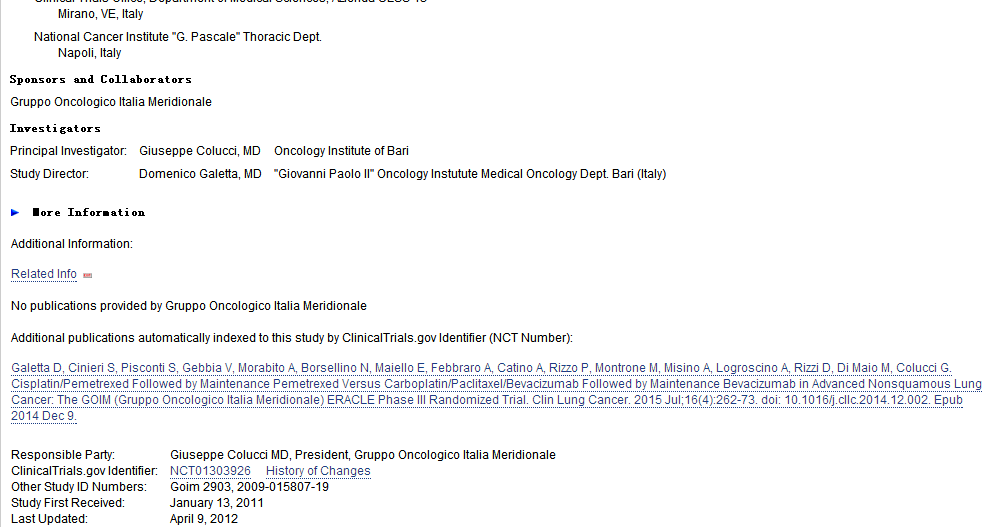

Supplement: S1 Protocol — (DOC) [file pone.0149247.s002.doc]
